# Nucleolar Proteins Suppress *Caenorhabditis elegans* Innate Immunity by Inhibiting p53/CEP-1

**DOI:** 10.1371/journal.pgen.1000657

**Published:** 2009-09-18

**Authors:** Laura E. Fuhrman, Ajay Kumar Goel, Jason Smith, Kevin V. Shianna, Alejandro Aballay

**Affiliations:** 1 Department of Molecular Genetics and Microbiology, Duke University Medical Center; Durham, North Carolina, United States of America; 2 Institute for Genome Sciences & Policy, Duke University, Durham, North Carolina, United States of America; Stanford University Medical Center, United States of America

## Abstract

The tumor suppressor p53 has been implicated in multiple functions that play key roles in health and disease, including ribosome biogenesis, control of aging, and cell cycle regulation. A genetic screen for negative regulators of innate immunity in *Caenorhabditis elegans* led to the identification of a mutation in NOL-6, a nucleolar RNA-associated protein (NRAP), which is involved in ribosome biogenesis and conserved across eukaryotic organisms. Mutation or silencing of NOL-6 and other nucleolar proteins results in an enhanced resistance to bacterial infections. A full-genome microarray analysis on animals with altered immune function due to mutation in *nol-6* shows increased transcriptional levels of genes regulated by a p53 homologue, CEP-1. Further studies indicate that the activation of innate immunity by inhibition of nucleolar proteins requires p53/CEP-1 and its transcriptional target SYM-1. Since nucleoli and p53/CEP-1 are conserved, our results reveal an ancient immune mechanism by which the nucleolus may regulate immune responses against bacterial pathogens.

## Author Summary

Innate immunity comprises a variety of defense mechanisms used by metazoans to prevent microbial infections. These nonspecific defense responses used by the innate immune system are governed by interacting and intersecting pathways that control not only immune responses but also longevity and responses to different stresses. Increasing evidence highlights the plurifunctional nature of the nucleolus, which appears to control various cellular processes involved in health and disease, from ribosome biogenesis to regulation of the cell cycle and the cellular stress response. We provide evidence indicating that the nucleolus suppresses innate immunity against bacteria by preventing the transcriptional activity of the tumor suppressor p53. We found that animals lacking nucleolar proteins are highly resistant to infections by bacterial pathogens. We also found that the activation of innate immunity by inhibition of nucleolar proteins requires potential immune effectors whose expression in response to stress is regulated by p53. Our study links the nucleolus, p53, and innate immunity against bacterial infections for the first time, and highlights a new mechanism that can potentially be exploited to alleviate bacterial infections.

## Introduction

The relatively simple innate immune system of the nematode *Caenorhabditis elegans* and the number of traits that facilitate genetic and genomic analysis using this organism have led to the discovery of several pathways that regulate innate immune responses to pathogen infections. Interestingly, many of the *C. elegans* innate immune pathways integrate responses to pathogens, oxygen, and various stresses [1],[2],[3],[4]. This suggests that multiple stress-sensing mechanisms are activated in response to bacterial infection. In addition to their role as ribosome factories, nucleoli also function in maturation of non-nucleolar RNAs or ribonucleoproteins, senescence and regulation of telomerase function, regulation of cell cycle, tumor suppressor and oncogene activities, and cell stress sensing [5],[6],[7],[8]. The stress-sensing function of the nucleolus, which involves the tumor suppressor p53, is one of its most important newly identified roles.

Although there are several ways in which p53 is regulated in mammals, changing the balance between its synthesis and degradation seems to be one of the most important. Under normal conditions, p53 is synthesized and then quickly degraded to maintain a very low level of the protein. The abundance of p53 is primarily regulated by the interplay of two proteins, MDM2 and ARF. In addition to binding to the transactivation domain of p53 [9],[10], MDM2 functions as an E3 ubiquitin ligase which targets p53 for export to the cytoplasm and/or proteasome-mediated degradation [11],[12],[13]. This auto-regulatory feedback loop likely acts to restrain p53 function in normal cells, in the absence of stress. ARF associates with MDM2 to inhibit the ubiquitination, nuclear export, and subsequent degradation of p53 [14],[15],[16]. The finding that ARF is primarily localized in the nucleolus [15],[17],[18] suggests that the nucleolus functions as a subnuclear compartment in which p53-activating proteins are sequestered in the absence of stress. Additionally, MDM2 has been shown to bind ribosomal protein L5 and 5S rRNA before export into the cytoplasm [19],[20], providing further evidence that nucleolar proteins are involved in the regulation of p53-regulating proteins. Even though there is no clear MDM2 orthologue in nematodes, the levels of active p53/CEP-1 are also known to be regulated at the translational and posttranscriptional levels in *C. elegans*. For example, GLD-1 controls the levels of p53/CEP-1 by binding to the 3-UTR of *cep-1* mRNA to repress its translation [21]. In addition, the Skp1/cullin/F-box (SCF) E3 ubiquitin ligase FSN-1 appears to negatively regulate endogenous CEP-1 protein phosphorylation levels [22].

In response to DNA damage, p53 levels rise as a consequence of activation of several kinases that phosphorylate the N-terminus of p53 preventing binding to MDM2. In response to cellular stress, such as DNA damage, heat shock, or hypoxia, p53 becomes stabilized and accumulates in the nucleus, leading to elevated transcriptional activity [23],[24],[25],[26]. In addition to the aforementioned stresses, it has been proposed that aberrant ribosome biogenesis may also cause “nucleolar stress” leading to stabilization of p53 in mice and human cells [27],[28],[29]. Disruption of the nucleolus, either by direct interference of ribosomal proteins [28],[29] or chemical inhibitors of ribosome biogenesis [29],[30] causes the release of p53-stabilizing proteins, and thus results in elevated levels of active p53. These findings indicate that the nucleolus may act as a stress sensor responsible for maintaining low levels of active p53 which become elevated upon impairment of nucleolar function.

Different genetic studies have led to the identification of several pathways involved in *C. elegans* innate immunity. However, while gene inactivation cripples innate immunity by affecting a variety of different pathways, only gene depletions that result in higher DAF-16 activity appear to promote innate immunity. DAF-16 is a FOXO transcription factor which regulates a wide variety of genes involved not only in immunity but also in stress-response, development, and longevity [3],[31],[32]. To identify genes that when mutated may enhance innate immunity without extending the life span of the nematodes, we undertook a comprehensive forward genetic analysis of nematodes exhibiting wild-type life span and enhanced resistance to the human Gram-negative pathogen *Salmonella enterica*. The study resulted in the isolation of a strain carrying a mutation in *nol-6*, which encodes a nucleolar RNA-associated protein. In addition, we demonstrate that RNAi-mediated depletion of *nol-6* as well as other nucleolar genes leads to an enhanced resistance to *S. enterica*-mediated killing that correlates with a reduction of pathogen accumulation. The results also show that animals deficient in *nol-6* are more resistant to infections by Gram-negative pathogen *Pseudomonas aeruginosa* and Gram-positive pathogen *Enterococcus faecalis*, indicating that nucleolar disruption activates immunity against different bacterial pathogens. Further studies indicate that nucleolar disruption through RNAi ablation of ribosomal genes results in an increased pathogen resistance that requires *p53*/*cep-1*. This study suggests that nucleolar disruption may be a mechanism by which *C. elegans* activates innate immunity against bacterial infection in a *p53*/*cep-1*-dependent manner.

## Results

### Forward genetic analysis to identify negative regulators of innate immunity

Until now, only gene depletions that result in higher activity of the FOXO transcription factor DAF-16 appear to promote innate immunity. DAF-16 is positively regulated by heat shock [33] and negatively regulated by the insulin-like receptor DAF-2 [34]. Thus, mutations in *daf-16* not only suppress the enhanced longevity of *daf-2* mutants, but also their enhanced resistance to pathogens [35],[36].

To identify genes involved in the regulation of innate immunity that do not affect the life span of *C. elegans*, we took a forward genetic approach. An important limitation of these types of studies is that resistant mutants would not be identified until much beyond the time frame for fertility, thus requiring the transfer of individual mutants in order to maintain each mutant line. This is tedious and time consuming, greatly reducing the number of animals that can be studied, particularly in the case of the various slow-killing pathogens of *C. elegans* [37]. For example, in the case of infections by *S. enterica*, hermaphrodite nematodes initially exposed to the pathogen need to be transferred each day to fresh plates to avoid losing track of these initial nematodes in the morass of progeny. Thus, we took advantage of the ability of *S. enterica* to cause a persistent colonization and luminal distension of the *C. elegans* intestine that correlate with the premature death of the animals [38],[39],[40].

**Figure 1:**
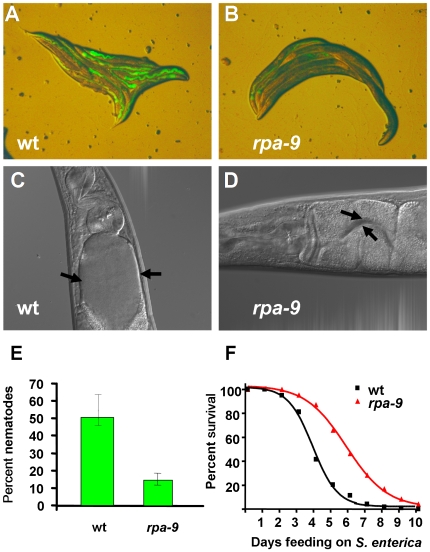
*rpa-9* mutants are resistant to both *S. enterica* accumulation and *S. enterica*-mediated killing. (A,B) Wild-type and *rpa-9* nematodes were fed *S. enterica*/GFP (SMO22) for 48 hours and then visualized using a MZ FLIII Leica stereomicroscope. (C,D) Wild-type and *rpa-9* nematodes were fed *S. enterica* (SL1344) for 48 hours and then visualized using a Zeiss Axioscope microscope. Arrows point to the border of the intestinal lumen. (E) Wild-type and *rpa-9* nematodes (p = 0.0206) were fed *S. enterica*/GFP (SMO22) for 48 hour, and the number of nematodes displaying GFP in the intestine was scored. Error bars represent SEM. (F) Wild-type and *rpa-9* nematodes (p<0.0001) were fed *S. enterica*, and the number of live versus dead animals was scored over time. For quantification of *S. enterica*/GFP accumulation, 66–116 animals were used. For each condition in the survival assays, 165–180 animals were used.

To identify *C. elegans* mutants which exhibit **r**educed **p**athogen **a**ccumulation (Rpa), EMS-mutagenized nematodes grown to one day old gravid adults on the laboratory food *Escherichia coli* strain OP50 were transferred to *S. enterica* strain SMO22 expressing GFP. A population of approximately 15,000 second generation mutants was screened for an Rpa phenotype after 48 hours of feeding on *S. enterica*/GFP. Out of 287 isolated *rpa* mutants, 43 mutants that generated progeny were further studied. Of these 43 *rpa* mutants, 9 mutants exhibited enhanced resistance to *S. enterica*-mediated killing (not shown). Five mutants exhibited stunted development and were therefore excluded from further analysis. Of the remaining 4 *rpa* mutants, *rpa-9* was chosen for mapping and further analysis based on the strength of its resistance to pathogen infection and lack of extended life span. Compared to wild-type nematodes, *rpa-9* mutant nematodes exhibit a reduced accumulation of *S. enterica*/GFP after 48 hours of feeding (Figures 1A, 1B, 1E, and S1). In addition, distension of the intestinal lumen 48 hours after initial exposure to *S. enterica* strain 1344 is completely suppressed in *rpa-9* mutants compared to wild type (Figure 1C and 1D). The reduced pathogen accumulation and intestinal distention of *rpa-9* mutants correlate with enhanced resistance to *S. enterica*-mediated killing (Figure 1F).

**Figure 2:**
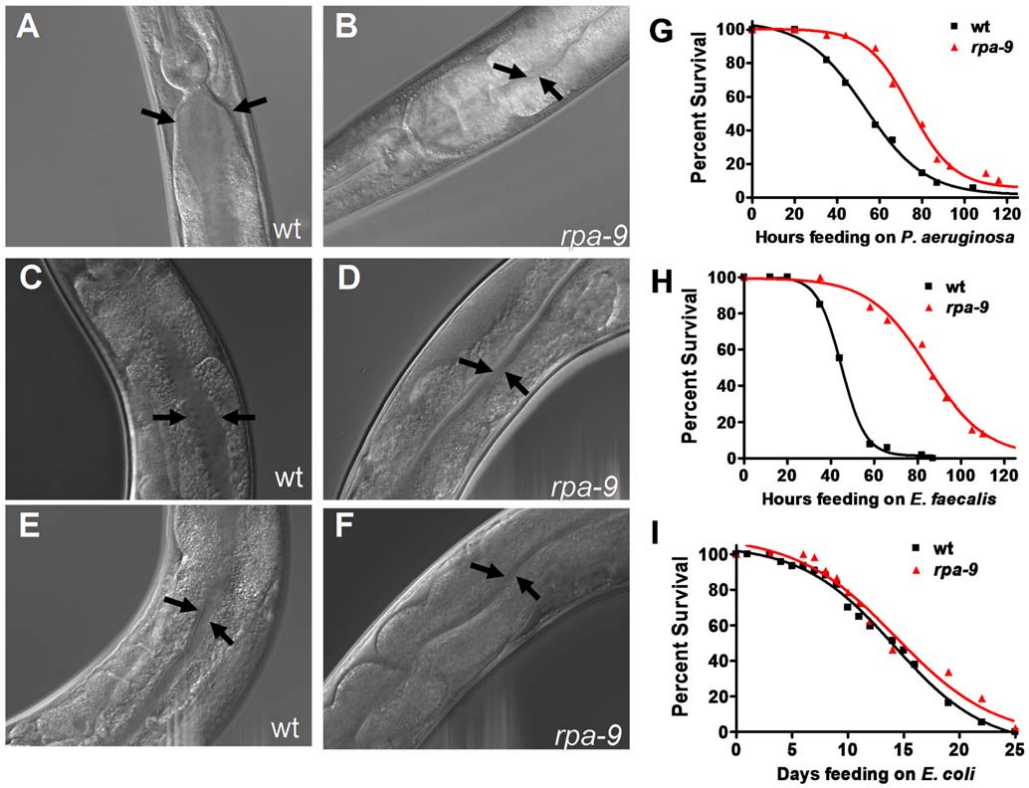
*rpa-9* mutants are resistant to pathogenic bacteria and exhibit wild-type longevity in the presence of *E. coli*. (A,B) Wild-type and *rpa-9* nematodes were fed *P. aeruginosa* (PA14) for 24 hours. (C,D) Wild-type and *rpa-9* nematodes were fed *E. faecalis* (OG1RF) for 48 hours. (E,F) Wild-type and *rpa-9* nematodes were fed *E. coli* (OP50) for 48 hours. (G) Wild-type and *rpa-9* nematodes (p<0.0001) were fed *P. aeruginosa* (PA14), and the number of live versus dead animals was scored over time. (H) Wild-type and *rpa-9* nematodes (p<0.0001) were fed *E. faecalis* (OG1RF), and the number of live versus dead animals was scored over time. (I) Wild-type and *rpa-9* nematodes (p>0.05) were fed *E. coli* (OP50), and the number of live versus dead animals was scored over time. The animals were visualized using a Zeiss Axioscope microscope, 40×. For each condition in the survival assays, 59–164 animals were used.

As shown in Figure 2, *rpa-9* exhibits resistance to accumulation and killing by other bacterial pathogens. Specifically, the intestinal lumen of *rpa-9* is not distended when infected with the Gram-negative pathogen *Pseudomonas aeruginosa* strain PA14 (Figure 2A and 2B) as well as the Gram-positive pathogen *Enterococcus faecalis* strain OG1RF (Figure 2C and 2D). In contrast, when fed relatively non-pathogenic *E. coli*, intestinal distension is absent in both wild-type nematodes and *rpa-9* mutants (Figure 2E and 2F). Consistent with previous studies indicating that intestinal distension and bacterial accumulation can correlate with nematode death [39],[41],[42], *rpa-9* exhibits enhanced resistance to killing by *P. aeruginosa* and *E. faecalis* (Figure 2G and 2H, respectively). These results suggest that, like *daf-2*, *rpa-9* acts as an inhibitor of innate immunity against different bacterial pathogens. However, unlike *daf-2*, the life span of *rpa-9* mutants is comparable to that of wild-type animals grown on *E. coli* lawns (Figure 2I). These results indicate that the resistance to pathogen infection of *rpa-9* mutants is not simply a consequence of an effect on life span extension and that immune mechanisms can be uncoupled from effects on aging.

**Figure 3:**
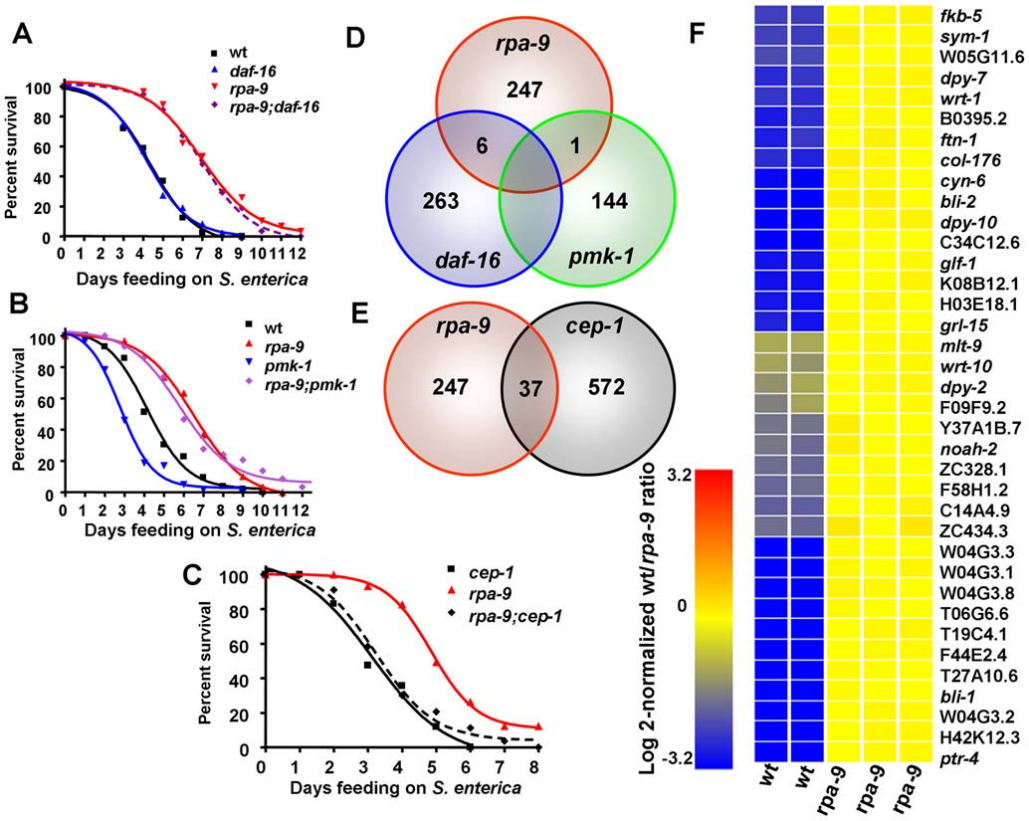
*rpa-9* mutation activates immunity against *S. enterica* in a p53/*cep-1*– dependent manner. (A) Wild-type, *rpa-9*, *daf-16(mu86)*, and *rpa-9;daf-16(mu86)* nematodes were fed *S. enterica*, and the number of live versus dead animals was scored over time. Wild type vs. *rpa-9*: p<0.0001. Wild type vs. *rpa-9;daf-16(mu86)*: p<0.0001. (B) Wild type, *rpa-9* mutant, *pmk-1(km25)* mutant, and *rpa-9;pmk-1(km25)* nematodes were fed *S. enterica*. Wild type vs. *rpa-9*: p<0.0001. Wild type vs. *pmk-1*: p<0.0001. Wild type vs. *rpa-9;pmk-1*: p<0.0001. *rpa-9* vs. *rpa-9;pmk-1*: p>0.05. (C) *rpa-9*, *cep-1(gk138)*, and *rpa-9;cep-1(gk138)* nematodes were fed *S. enterica*, and the number of live versus dead animals was scored over time. *cep-1* vs. *rpa-9* p<0.001. *cep-1* vs. *rpa-9;cep-1(gk138)* p>0.05. Since *cep-1(gk138)* nematodes exhibit an Egl phenotype, the animals that die from matricide were censored. (D) Venn diagram of genes that are upregulated in *rpa-9* mutant and positively regulated by *daf-16* or *pmk-1*. (E) Venn diagram of genes upregulated in *rpa-9* mutants and induced upon UV radiation in a *cep-1*–dependent manner. (F) Cluster of *cep-1* regulated genes that are upregulated in *rpa-9*. For each condition in the survival assays, 57–61 animals were used.

In addition to enhanced resistance to pathogen infection, *rpa-9* nematodes also exhibit temperature sensitive reduced fertility and larval lethality of progeny when raised at the restrictive temperature of 25°C. The study of sterile mutants that are temperature sensitive demonstrated that sterility may result in enhanced resistance to pathogen through a DAF-16-dependent mechanism [43]. Since the *C. elegans* infections are performed at 25°C, it is conceivable that the enhanced resistance to pathogens of *rpa-9* mutants is a consequence of the reduced fertility at 25°C. However, *rpa-9* mutants are also resistant to pathogen-mediated killing when the infections are performed at the permissive temperatures of 20°C and 15°C (Figure S2), indicating that their enhanced resistance to pathogens is not simply a consequence of reduced fertility. DAF-16 activation by reduction of *daf-2* function causes not only resistance to pathogen infection, but also entry into an alternative larval stage and dramatic increase in longevity when the animals are grown on the laboratory food *E. coli* [44],[45],[46]. In contrast to *daf-2* mutants, *rpa-9* mutants exhibit a life span that is comparable to that of wild-type animals when grown on plates containing *E. coli* (Figure 2I), suggesting that the enhance resistance to pathogens of *rpa-9* mutants is caused by a mechanism that is independent of the DAF-16 effects on longevity. Consistent with this idea, a *daf-16* mutation known to suppress the enhanced resistance to pathogen phenotype of *daf-2* mutants does not suppress the enhanced resistance to *S. enterica* of *rpa-9* mutants (Figure 3A).

### Derepression of CEP-1 transcriptional activity in *rpa-9* mutants activates immunity against *S. enterica*

To provide insight into the mechanism underlying the enhanced immunity of *rpa-9* mutants, we utilized gene expression microarrays to find clusters of genes upregulated or downregulated in *rpa-9* mutants relative to wild-type animals grown on *S. enterica*. Overall, the microarray data show poor overlap with genes previously known to be regulated by pathways involved in *C. elegans* innate immunity (Figure 3D and Table S1). Out of 247 upregulated genes in *rpa-9* nematodes relative to wild-type nematodes, only seven genes have been linked to innate immune pathways in *C. elegans*. As shown in Figure 3D, only six upregulated genes are regulated by DAF-16 and one upregulated gene is regulated by the *C. elegans* p38 MAP kinase, PMK-1, which like DAF-16, plays a crucial role in innate immunity [38],[47],[48],[49],[50]. The lack of major enrichment in DAF-16 and PMK-1-regulated genes is consistent with the lack of suppression of the enhanced resistance to *S. enterica* of *rpa-9* nematodes by loss of DAF-16 or PMK-1 (Figure 3A and 3B).

The microarray data show that the *rpa-9* mutation results in a significant enrichment in genes regulated by the *C. elegans* homologue of p53, CEP-1, which plays a role in apoptosis, meiosis, and stress resistance [21],[51],[52],[53],[54] (Figure 3E, 3F, Table S1, Table S2, and Table S3). Quantitative real-time polymerase chain reaction (qRT-PCR) confirmed the up-regulation of CEP-1-regulated genes in *rpa-9* mutants (Figure S3), suggesting that higher CEP-1 activity is responsible for the enhanced immunity against *S. enterica* of *rpa-9* animals. Thus, we studied whether a loss-of-function mutation in *cep-1(gk138)* nematodes [55] suppresses the enhanced resistance to *S. enterica*-mediated killing of *rpa-9* nematodes. Since *cep-1(gk138)* nematodes exhibit an Egl phenotype, the animals that die from matricide were censored. As shown in Figure 3C, *cep-1(gk138)* mutation suppresses the enhanced resistance to *S. enterica*-mediated killing of *rpa-9* nematodes, indicating that higher CEP-1 activity is required for activation of immunity against *S. enterica*.

### Mutation or RNAi inhibition of *nol-6* enhances *C. elegans* innate immunity

Whole genome sequencing of *rpa-9* mutants and analysis of the RFLP-SNP mapped 109-kilobase region revealed a single mutation within this region. The *rpa-9*/*nol-6(ac1)* allele is a G to A substitution in the third exon of the *C. elegans* gene *nol-6* resulting in a glycine to glutamic acid substitution at amino acid position 151 (Figure S4). Since glycine is the smallest of the amino acids and can be either positively or negatively charged depending upon the environment, it is likely that substitution with a large, highly polar amino acid such as glutamic acid will alter the folding pattern of the protein and potentially hinder its function.

**Figure 4:**
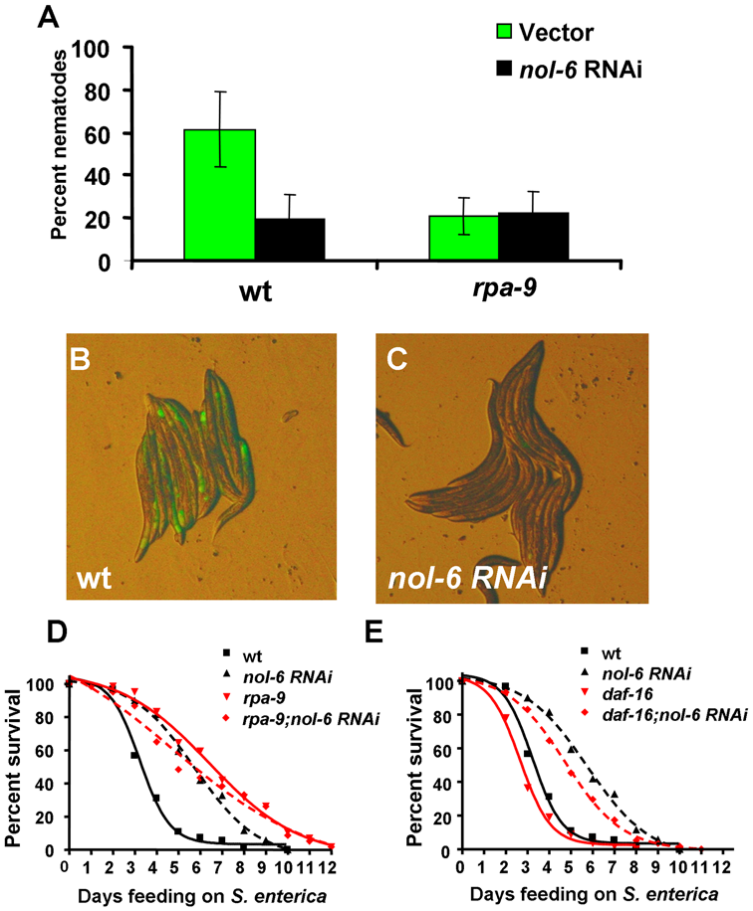
*nol-6* acts as a suppressor of immunity against *S. enterica*. (A) Wild-type and *rpa-9* nematodes grown on dsRNA for vector control or dsRNA for *nol-6* were fed *S. enterica*/GFP for 48 hours, and the number of animals displaying GFP within the intestine was scored. Vector vs. *nol-6* RNAi: p = 0.0268. *rpa-9* vs. *rpa-9;nol-6* RNAi: p = 0.8982. Error bars represent SEM. (B,C) Wild-type nematodes grown on dsRNA for vector control or dsRNA for *nol-6* were fed *S. enterica*/GFP for 48 hours and then visualized using a MZ FLIII Leica stereomicroscope. (D) Wild-type and *rpa-9* nematodes grown on dsRNA for vector control or dsRNA for *nol-6* were fed *S. enterica*, and the number of live versus dead animals was scored over time. Wild type vs. *nol-6 RNAi*: p<0.0001. (E) Wild type nematodes and *daf-16(mu86)* nematodes grown on dsRNA for vector control or dsRNA for *nol-6* were fed *S. enterica*. Vector vs. *nol-6 RNAi*: p<0.0001. *daf-16(mu86)* vs. *nol-6 RNAi*: p<0.0001. Vector vs. *daf-16(mu86)*: p = 0.0649. *nol-6 RNAi* vs. *daf-16(mu86);nol-6 RNAi*: p = 0.0173. For quantification of *S. enterica*/GFP accumulation, a total of 127–176 animals were used for each condition. For survival assays, 54–61 animals were used for each condition.

To study whether *nol-6* acts as a suppressor of innate immunity, we first compared *S. enterica* intestinal accumulation of wild-type nematodes to that of nematodes in which *nol-6* gene expression was depleted by RNAi. As shown in Figure 4A–4C, *nol-6* RNAi in wild-type nematodes results in a significant decrease in the percentage of nematodes exhibiting intestinal accumulation of *S. enterica*/GFP 48 hours after the infection. Additionally, the number of intestinal *S. enterica* colony forming units in *nol-6* RNAi and *rpa-9(ac1)* nematodes is lower than that in control animals (Figure S5). Consistent with the idea that the enhanced resistance to pathogen infection of *rpa-9* animals is due to a mutation in *nol-6*, *nol-6* RNAi enhances nematode resistance to *S. enterica*-mediated killing (Figure 4D). Furthermore, *nol-6* RNAi in *rpa-9* mutant nematodes results in no significant change in intestinal accumulation of *S. enterica*/GFP, as expected if *rpa-9* is allelic to *nol-6* (Figure 4A). RNAi-mediated depletion of *nol-6* in *rpa-9* nematodes results in no significant change in survival further supporting the idea that *rpa-9* is allelic to *nol-6* (Figure 4D). Consistent with the observation that *daf-16(mu86)* does not suppress the enhanced resistance to *S. enterica* of *rpa-9* mutants (Figure 3A), *daf-16(mu86)* does not suppress the enhanced resistance to *S. enterica* of *nol-6* RNAi nematodes (Figure 4E). In addition, *nol-6* RNAi phenocopies the reduced fertility of *rpa-9* mutants when raised at 25°C (Figure S6), providing further evidence that *rpa-9* is allelic to *nol-6*.

### Loss of nucleolar proteins enhances *C. elegans* resistance to *S. enterica*

**Figure 5:**
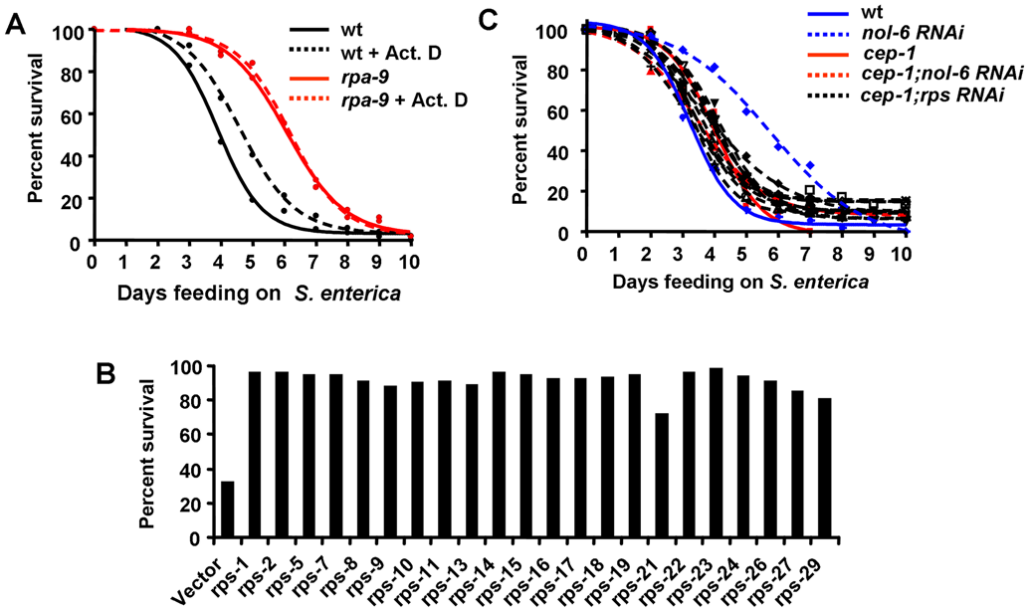
Nucleolar protein knockdown activates immunity against *S. enterica* in a p53/*cep-1*–dependent manner. (A) Wild type and *rpa-9* nematodes were fed *S. enterica* on plates containing either 0.005 µg/mL actinomycin D or buffer. Wild type vs. Wild type+Act. D: p = 0.0261. (B) Wild type nematodes were grown on dsRNA for vector control or dsRNA for *rps* genes, and the number of living nematodes was scored after five days of feeding on *S. enterica*. Differences between vector and *rps RNAi* was statistically significant in all cases, p<0.0001. (C) Wild type and *cep-1(gk138)* nematodes were grown on dsRNA for vector control, dsRNA for *nol-6*, or dsRNA for *rps* genes, and the number of live versus dead animals was scored over time. Wild type vs. *nol-6 RNAi*: p<0.0001. Wild type vs. *cep-1(gk138);nol-6 RNAi*: p>0.05. Since *cep-1(gk138)* nematodes exhibit an Egl phenotype, the animals that die from matricide were censored. For each condition, 57–61 animals were used.

*C. elegans nol-6* encodes a **n**ucleolar **R**NA **a**ssociated **p**rotein (NRAP) that is conserved across eukaryotic organisms and involved in early stages of ribosome biogenesis [56]. The first step of generating a ribosome subunit requires the initial transcription of rDNA genes by RNA polymerase I (Pol-I). Inhibition of Pol-I by actinomycin D, an inhibitor of ribosome biogenesis [57],[58], leads to an enhanced resistance to *S. enterica*-mediated killing in wild-type nematodes without significantly affecting *S. enterica* virulence (Figure 5A). However, actinomycin D treatment in *rpa-9* mutant nematodes has no effect (Figure 5A), suggesting that the ribosomal stress caused by the mutation cannot be further enhanced by drug treatment. Even though the nucleoli of *S. enterica*-infected animals are slightly larger than that of animals grown on *E. coli* and the nucleoli of *rpa-9* mutants are also larger than the nucleoli of wild type nematodes when fed *S. enterica* (Figure S7), the small changes observed suggest that the overall structure of the nucleoli is not extensively affected.

In order to elucidate whether general disruption of ribosomal proteins can lead to enhanced pathogen resistance, we used RNAi to knock down individual ribosomal protein subunit (*rps*) genes and the percentage of live nematodes was determined five days after the infection by *S. enterica*. As shown in Figure 5B, five days after the infection only 30% of control nematodes remained alive, while 72% to 98% of nematodes in which individual *rps* genes were depleted by RNAi remained alive. To address whether the germline may affect the enhanced resistance to pathogen infection of *nol-6* or *rps* RNAi nematodes, RNAi was performed in germline-deficient animals *glp-4(bn2)*. Inhibition of *nol-6* or *rps* genes by RNAi enhances the median survival of *glp-4(bn2)* nematodes infected with *S. enterica* by 25–33% (Table S4), indicating that loss of ribosomal proteins activates innate immunity even in the absence of a fully developed germline. Since loss of ribosomal proteins enhances resistance of wild type animals to *S. enterica*-mediated killing by 41–64%, it is possible that the germline responds to nucleolar stress and contributes to the activation of innate immunity in wild-type animals. Taken together, these results provide the first indication that the ribosome acts as a negative regulator of innate immunity and that reduced ribosomal function by mutation or RNAi boosts innate immunity.

### Enhanced immunity by depletion of *nol-6* and *rps* genes requires *p53*/*cep-1*

Elevated p53 transcriptional activity in response to various cellular stresses such as DNA damage, heat shock and hypoxia has previously been reported [23],[24],[25],[26]. In addition, aberrant ribosome biogenesis can lead to stabilization of p53 in mice and human cells [27],[28],[29]. Therefore, we hypothesized that higher p53 activity, as a consequence of aberrant ribosome biogenesis and nucleolar stress, in *nol-6* and *rps* RNAi animals results in enhanced resistance to *S. enterica*. To test this hypothesis, we compared *S. enterica*-mediated killing of loss-of-function *cep-1(gk138)* nematodes [55] to that of *cep-1(gk138)* nematodes in which *nol-6* and *rps* RNAi gene expression was depleted by RNAi. As shown in Figure 5C, *cep-1* mutation suppresses not only the enhanced resistance to *S. enterica*-mediated killing of *nol-6* RNAi nematodes but also that of *rps* RNAi nematodes, indicating that derepression of CEP-1 transcriptional activity by *nol-6* or *rps* RNAi activates immunity against *S. enterica*. After development, CEP-1 is highly expressed in the pharynx [51], which we have recently demonstrated plays a key role in *C. elegans* immunity against *S. enterica* [2],[59]. Interestingly, not only CEP-1, but also PMK-1 and DAF-16 appear to be required for the enhanced resistance to *P. aeruginosa* of *rpa-9* nematodes (Figures S8, S9, and S10). These results are consistent with previous studies that showed that different mechanisms mediate innate immunity to *S. enterica* and *P. aeruginosa* [59],[60].

**Figure 6:**
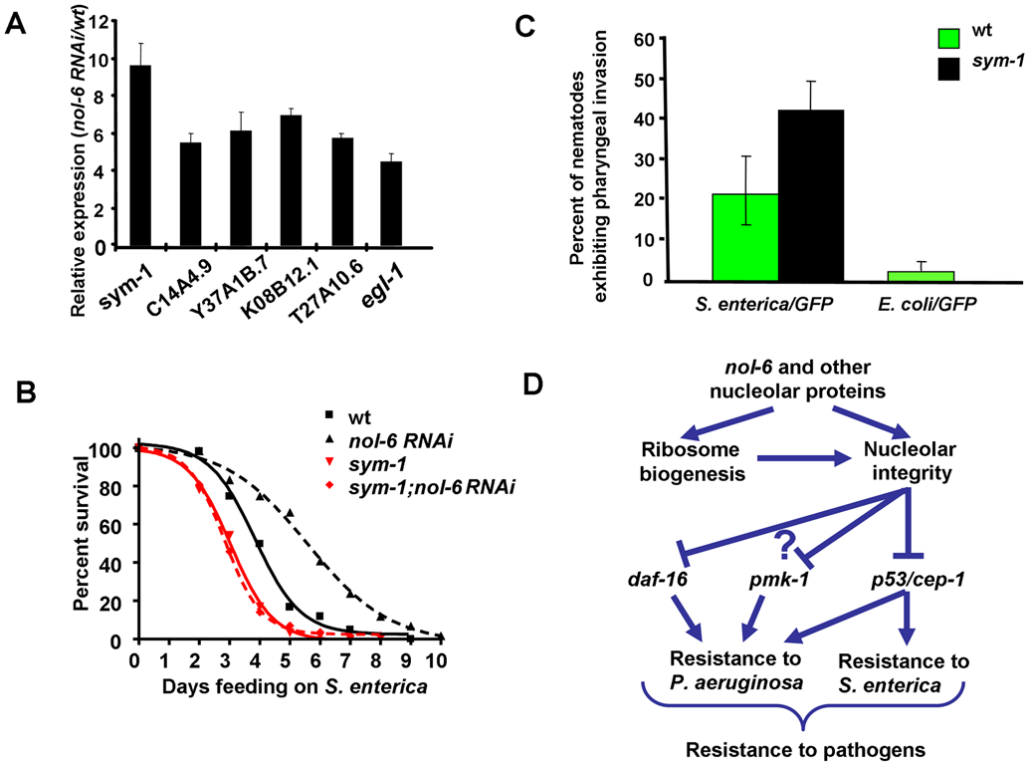
Derepression of CEP-1 transcriptional activity by *nol-6* RNAi activates immunity against *S. enterica.* (A) Quantitative RT-PCR analysis of 7 *cep-1*–dependent genes in nematodes grown on dsRNA for vector control or dsRNA for *nol-6*. Data were analyzed by normalization to pan-actin *(act-1,-3,-4)* and relative quantification using the comparative-cycle threshold method. Student's exact *t* test indicates that differences among the groups are significantly different; bar graphs correspond to mean±SEM (n = 3). (B) Wild type and *sym-1(mn601)* nematodes grown on dsRNA for vector control or dsRNA for *nol-6* were fed *S. enterica*, and the number of live versus dead animals was scored over time. Vector vs. *sym-1(mn601)*: p = 0.0002. For each condition, 60 animals were used. This experiment was performed in duplicate. (C) Wild type and *sym-1(mn601)* nematodes were fed *S. enterica/GFP* or *S. enterica/GFP* for 48 hours, and the percentages of worms exhibiting pharyngeal invasion of *S. enterica* were quantified. Wild type vs. *sym-1(mn601) S. enterica/GFP*: p<0.0001. For each condition, approximately 100 animals were used. (D) Mechanism by which disruption of the nucleolus may lead to enhanced resistance to pathogen through the activation of CEP-1. Inhibition of *nol-6* and other nucleolar proteins via RNAi or mutation disrupts nucleolar integrity leading to an upregulation of CEP-1-dependent transcription and an increase in resistance to both *S. enterica* and *P. aeruginosa*. Disruption of the nucleolus may also lead to enhanced resistance to *P. aeruginosa* through the activity of PMK-1 and DAF-16.

Consistent with the idea that higher CEP-1 activity is responsible for the enhanced immunity against *S. enterica* of NOL-6-deficient animals, the microarray data show a significant enrichment in CEP-1-regulated genes in *rpa-9* mutants (Figure 3E and Table S1). The expression analysis of five studied genes that belong to the cluster of CEP-1-regulated genes that are induced in *rpa-9* mutants (Figure 3F) shows that they are also upregulated in *nol-6* RNAi nematodes compared to control wild-type nematodes (Figure 6A). An additional known CEP-1 target, *egl-1* [61],[62],[63] was also found to be upregulated in *nol-6* RNAi nematodes compared to vector control wild-type nematodes (Figure 6A). Taken together, these results suggest that higher CEP-1 activity is responsible for the enhanced resistance to *S. enterica*-mediated killing in animals with impaired ribosomal function due to mutation or RNAi of *nol-6*, and suggest that the nucleolus suppresses innate immunity in a CEP-1-dependent manner (Figure 6D).

The most highly upregulated gene in *rpa-9* or *nol-6* RNAi animals was the leucine rich repeat (LRR) encoding gene *sym-1*, which is one of twelve genes that are signature of *C. elegans* response to infections by different pathogens, including *S. enterica* (Table S2, and Aballay and Tenor unpublished data). To investigate the importance of *sym-1* during *S. enterica* infection, we compared the survival of *sym-1(mn601)* null mutants [64] with that of wild-type nematodes. Indeed, *sym-1(mn601)* mutant nematodes exhibit enhanced susceptibility to *S. enterica*-mediated killing (Figure 6B). In addition, the *sym-1(mn601)* mutation completely suppresses the resistance phenotype conferred by *nol-6* RNAi (Figure 6B). It should be noted that a significant number of *sym-1(mn601)* nematodes die from matricide during the early time point of the assay. However, when matricide is censored, *nol-6* RNAi still fails to enhance resistance to *S. enterica*-mediated killing in *sym-1(mn601)* animals (Figure S11). In addition to providing protection from *S. enterica*-mediated killing, *sym-1* is also required to prevent *S. enterica* invasion of the pharynx (Figure 6C). Taken together, these data indicate that higher CEP-1 activity results in the expression of genes important for proper immune response to the bacterial pathogen *S. enterica*.

## Discussion

Increasing evidence indicates that the nucleolus plays a role as a coordinator of cellular stress responses by regulating the activity of p53. However, the relationship between nucleolar proteins and p53 in response to bacterial infections has not been studied. In this study, we provide evidence indicating that nucleolar proteins suppress innate immunity against bacteria by preventing the transcriptional activity of p53. Animals lacking NOL-6 and other nucleolar proteins were found to be resistant to infections by bacterial pathogens. Importantly, whole-genome microarray analyses and subsequent qRT-PCR studies demonstrated that inhibition of the nucleolar protein NOL-6 by mutation or RNAi results in higher activity of the *C. elegans* homologue of p53, CEP-1. Furthermore, we found that CEP-1 and SYM-1, which is induced by UV irradiation in a CEP-1-dependent manner [55], are required for the enhanced resistance to pathogen infection of animals lacking NOL-6. The results indicate that nucleolar stress, which may be caused by loss of nucleolar proteins, pathogen infection, or UV irradiation, enhances innate immunity by activating the transcriptional activity of CEP-1.

To date, the only identified suppressor of innate immunity is DAF-2, which acts through inhibition of the FOXO transcription factor DAF-16 [35]. Until now, all the known suppressors of *C. elegans* innate immunity act through DAF-16-dependent mechanisms and, as a consequence, increase *C. elegans* longevity [35],[43]. Therefore, enhanced resistance to pathogen infection by loss of nucleolar proteins represents the first mechanism by which enhanced innate immunity does not result in enhanced longevity in *C. elegans*. Even though DAF-16 is not required for the enhanced resistance to *S. enterica*-mediated killing of *rpa-9* mutants (Figure 3A), it is required for the enhanced resistance to *P. aeruginosa*-mediated killing (Figure S10). These results indicate that different mechanisms mediate innate immunity to *S. enterica* and *P. aeruginosa*. Further analysis will be required to understand the role of DAF-16 in the enhanced resistance to *P. aeruginosa* by loss of nucleolar proteins.

Nucleolar RNA-associated proteins like NOL-6 are largely conserved across eukaryotic organisms and have been shown to associate closely with condensed chromosomes during mitosis, suggesting an involvement in ribosomal RNA (rRNA) processing during the early stages of ribosome biogenesis [56]. Our results show that disruption of ribosomes via treatment with actinomycin D or by RNAi-mediated knockdown of individual ribosomal protein subunit (*rps*) genes leads to an enhanced resistance to *S. enterica* infection. Ribosomal proteins are required for proper germline development [65],[66] and CEP-1 plays a role in stress-induced germline apoptosis in *C. elegans* [67]. To address whether the germline may affect the enhanced resistance to pathogen infection of *nol-6* or *rps* RNAi nematodes, RNAi was performed in germline-deficient animals *glp-4(bn2)*. Inhibition of *nol-6* or *rps* genes by RNAi enhances the median survival of *glp-4(bn2)* nematodes infected with *S. enterica* by 25–33% (Table S4), indicating that loss of ribosomal proteins activates innate immunity by a mechanism that does not require CEP-1 expression in the germline.

The nucleolus has been linked to the regulation of p53 via sequestration of p53-activating proteins [15],[17],[18]. In addition, it has been demonstrated that nucleolar disruption due to the effects of DNA-damaging agents is the cause of p53 accumulation [29]. These findings further support the function of the nucleolus as a stress sensor responsible for maintaining low levels of active p53 which become elevated upon impairment of nucleolar function. Our studies show that disruption of the *C. elegans* nucleolar protein NOL-6 leads to increased transcriptional levels of CEP-1-regulated genes and a significant enhanced resistance to the bacterial pathogen *S. enterica*. A comparison of genes that are misregulated in *rpa-9* mutant animals and genes that require *cep-1* for proper regulation following ultraviolet irradiation [55] revealed a significant overlap between the two gene sets (Figure 3E and Table S1). These findings suggest that disruption of the nucleolus by mutation in *nol-6* leads to enhanced resistance to *S. enterica* through increased p53 activity. Further studies show that the enhanced resistance to *S. enterica* imposed by *rpa-9* mutation or *nol-6* (RNAi) is suppressed in a *cep-1(gk138)* mutant background (Figures 3C and 5C), indicating that an increase in CEP-1 activity is required for the protective effect.

The suppression of the enhanced resistance to *S. enterica* of *rpa-9* and *nol-6* RNAi animals by *cep-1* mutation suggests that nucleolar disruption by loss of nucleolar proteins results in the activation of a CEP-1-dependent immune response. Consistent with this idea, *sym-1*, which has been shown to be regulated by CEP-1 in response to UV irradiation [55] and is the most highly induced gene in *rpa-9* and *nol-6* RNAi animals, was found to be required for the enhanced immunity of *nol-6* RNAi nematodes (Figure 6B and 6C). Like Toll receptors that function in both development and immunity in *Drosophila*, SYM-1 may regulate the two processes in *C. elegans*. Although *sym-1(mn601)* mutation does not cause a discernible phenotype, in combination with mutations that affect a key regulator of alternative splicing it results in deficient muscle attachment to the cuticle during development [64]. Thus, subtle developmental deficiencies due to lack of *sym-1* may weaken *C. elegans*, increasing its susceptibility to pathogen infection. Interestingly, *sym-1* encodes a leucine rich repeat (LRR) which is found in the majority of pattern recognition receptors involved in innate immunity. LRRs, are found in proteins ranging from plant resistance (*R*) genes [68] to Toll or Toll-like receptors in species ranging from insects to mammals [69]. Recent work indicates that TOL-1 is required for *C. elegans* immunity against *S. enterica* and for the correct expression of *abf-2*, an antimicrobial peptide encoding gene, and *hsp16.41* [59], which is part of the heat shock pathway required for immunity in *C. elegans* [36],[70]. Additionally, a recent screen of candidate LRR receptors in *C. elegans* has led to the identification of FSHR-1 as an essential component of innate immunity [71]. Further studies will be required to address whether the LRR-containing proteins, TOL-1, FSHR-1, and SYM-1 function as pathogen recognition receptors or play different roles in *C. elegans* defense against bacterial pathogens.

In summary, using forward and reverse genetics we have identified a new mechanism by which innate immunity is regulated. Our results provide evidence that nucleolar proteins and p53/CEP-1 transcriptional activity play a role in defense response against infections by bacterial pathogens. In animals lacking nucleolar proteins and infected with bacterial pathogens, nucleolar stress leads to the activation of a p53/CEP-1-mediated immune mechanism. Given the conserved functions of nucleoli and p53/CEP-1, our findings provide a mechanism by which the nucleolus may regulate antibacterial responses across metazoans.

## Materials and Methods

### *C. elegans* strains

*C. elegans* strains were cultured and maintained using standard procedures [72]. The following strains were kindly provided by the *Caenorhabditis* Genetics Center (University of Minnesota, St. Paul, Mn, USA): wild-type var. Bristol (N2), Hawaiian mapping strain (CB4856), *daf-16(mu86)*, *cep-1(gk138)*, *sym-1(mn601)*, *pmk-1(km25)*, and *glp-4(bn2)*. *rpa-9/nol-6(ac1)* animals were generated in this study and backcrossed to wild type 4 times before analysis.

### Mutagenesis

EMS (ethane methyl sulfonate) mutagenesis was performed as previously described [73]. Briefly, the wild-type strain Bristol N2 was mutagenized with 50 mM EMS for 4 hours at 20°C. This is expected to generate ∼220 G/C→A/T transition mutations per haploid genome, ∼50 of which cause amino acid mutations in protein coding genes [73],[74]. Mutagenized progeny were harvested and allowed to self-fertilize in order to fix induced mutations.

### Isolation of *rpa C. elegans* mutants

Mutagenized nematodes were grown to one day old gravid adults on *E. coli* strain OP50 [72] before transfer to *S. enterica/*GFP strain SMO22 [75] for 48 hours. Nematodes were visualized using a Leica MZ FLIII fluorescence stereomicroscope and mutants which displayed little or no GFP within the intestine were isolated and propagated on individual plates.

### Microscopy

*C. elegans* strains were grown exactly as described for *C. elegans* killing assays. For *S. enterica*, *E. faecalis* and *E. coli*, *C. elegans* were fed the bacteria for 48 hours before being harvested and transferred to an agar pad on microscope slides in sodium azide for visualization. For *P. aeruginosa*, *C. elegans* were exposed for 24 hours prior to visualization. *C. elegans* were imaged under a 40× oil immersion objective and processed with a Zeiss Axioscope epifluorescence microscope equipped with a Hamimatsu CCD camera and processed with Axiovision v3.0 imaging software.

### *C. elegans* killing assays

*C. elegans* wild-type Bristol N2 animals and mutants were maintained as hermaphrodites at 20°C, grown on modified nematode growth medium (NGM) agar plates, and fed with *E. coli* strain OP50 as described [72]. *S. enterica* strain SL1344 [76], *Pseudomonas aeruginosa* strain PA14 [41], and *Enterococcus faecalis* strain OG1RF [77] cultures were grown in Luria–Bertani (LB) broth at 37°C. *S. enterica* and *P. aeruginosa* bacterial lawns used for *C. elegans* killing assays were prepared by spreading 25 µl of an overnight culture of bacteria on modified NGM agar (0.35% instead of 0.25% peptone) in plates 3.5 cm in diameter. *E. faecalis* bacterial lawns were prepared by spreading 25 µl of an overnight culture on brain-heart infusion (BHI) agar on plates 3.5 cm in diameter. Nematodes were scored and transferred once a day to fresh plates. Nematodes were considered dead when they failed to respond to touch. For killing assays involving actinomycin D, 0.5 µg/mL actinomycin D (Sigma-Aldrich) was added to the NGM agar and plates were inoculated with 25 µl of an overnight culture of *S. enterica*. All assays were performed at 25°C unless otherwise noted. All the experiments were performed in triplicate unless otherwise indicated.

### Quantification of intestinal accumulation of *S. enterica*/GFP

*C. elegans* strains were grown exactly as described for *C. elegans* killing assays. After 48 hours of feeding on *S. enterica*/GFP strain Smo22, nematodes were transferred to *E. coli* strain OP50 and visualized using a Leica MZ FLIII fluorescence stereomicroscope. In each case, graphs represent combined data from three independent experiments. Differences between bar graphs were considered statistically significant when p<0.05 using a two-tailed t-test in PRISM 4.0.

### Generation of Hawaiian recombinant mutant strains

Five *rpa-9* hermaphrodites were placed with 10 Hawaiian CB4856 males for mating at 20°C overnight. After 24 hours, males were removed and hermaphrodite *rpa-9* nematodes were isolated to separate plates. Thirty F1 progeny were isolated from a single successful mating and allowed to self-fertilize. Twelve F2 progeny were collected from each F1 progeny (a total of 360) and were allowed to egg-lay on two sets of plates. One set of plates was maintained at 20°C and kept as stocks. The second set of plates was transferred to 25°C. Because the Rpa phenotype is not 100% penetrant, cross progeny which displayed the temperature sensitive larval lethal phenotype of *rpa-9* mutants were isolated and screened for enhanced resistance to *P. aeruginosa*. Recombinants that displayed the temperature sensitive larval lethality also displayed enhanced resistance to *P. aeruginosa* (data not shown). 96 positive recombinants were used for genotyping.

### Primer design for RFLP analysis

RFLP-SNPs and surrounding sequences were obtained from the *C. elegans* SNP database (http://genome.wustl.edu/genome/celegans/celegans_snp.cgi). Primers were designed using the Primer 3 program (http://frodo.wi.mit.edu/cgi-bin/primer3/primer3_www.cgi). Oligonucleotides were synthesized and HPLC purified by MWG Biotech (Ebersberg, Germany).

### PCR conditions for RFLP analysis

DNA lysates were generated by suspending 50 nematodes in 100 µl lysis buffer (50 mM NaCl, 10 mM Tris-Cl pH 7.5, 2.5 mM MgCl2, 0.45% Tween 20, 0.01% gelatin, 0.2 mg/ml proteinase K) and lysed at 65°C for one hour. 2 µl of this crude lysate was used as the PCR template. The PCR reactions contained 100 µM of each the forward and reverse primer, 125 µM dNTPs, 0.5 U Choice-*Taq* DNA polymerase and 1× Choice-*Taq* PCR buffer (Denville Scientific Inc., Metuchen, NJ, USA), in a 25 µl total volume. All reactions were performed in 96-well PCR plates sealed with an adhesive cover as follows: initial denaturation at 95°C for 2 minutes, followed by 30 cycles of 95°C denaturation for 30 seconds, 55°C annealing for 30 seconds, 72°C extension for 30 seconds, and ending with a 7 minute final extension at 72°C.

### Restriction digest conditions

All restriction digests were performed using New England BioLabs, Inc (NEB) enzymes and the recommended corresponding NEB buffer in a total reaction volume of 20 µl. A 2× digestion master mix was made as follows: 2× NEB buffer, 2× BSA, 3 U NEB enzyme to a total volume of 1 mL. 10 µl of each PCR reaction was added to 10 µl of 2× digestion master mix in a new 96-well plate and incubated at the appropriate temperature for 2 hours. Digested PCR fragments were resolved on 2% agarose gels and visualized with ethidium bromide staining and the genotype determined. As the region containing *rpa-9* became narrower, SNPs which did not disrupt a restriction site were sequenced using traditional Sanger sequencing techniques for analysis.

### High-throughput mapping of *rpa-9* identifies a missense mutation in *nol-6*

Mutant *rpa-9* was crossed to the Hawaiian SNP mapping strain CB4856 and approximately four hundred second generation cross progeny were isolated and screened for an *rpa-9* phenotype. DNA from a total of 96 *rpa-9* positive recombinant progeny was isolated and used for RFLP-SNP mapping. SNP data from the two most informative recombinants identified a 109-kilobase region on chromosome II between positions 1,407,386 and 1,516,505 containing the *rpa-9* mutation (Figure S4).

Recently, whole genome sequencing has been validated as a powerful new method of detecting lesions caused by chemical mutagens in *C. elegans* [78],[79],[80]. Whole genome sequencing of *rpa-9* mutants and analysis of the RFLP-SNP mapped 109-kilobase region revealed a single mutation within this region. This G to A mutation occurred in the third exon of the *C. elegans* gene *nol-6* resulting in a glycine to glutamic acid substitution at amino acid position 151. Since glycine is the smallest of the amino acids and can be either positively or negatively charged depending upon the environment, it is likely that substitution with a large, highly polar amino acid such as glutamic acid will alter the folding pattern of the protein and potentially hinder its function.

In an attempt to rescue the mutation by transgenic complementation, different constructs carrying *nol-6* cDNA or genomic *nol-6* were used to express wild-type *nol-6* in *rpa-9* mutants. In the cases where the reporter gene *gfp* was used, GFP expression was observed in embryos and larvae of F1 animals. However, GFP expression steadily declined during development and was not observed in the progeny. Overall, animals expressing different transgenes failed to produce progeny, suggesting potential toxicity of the transgene. Because microinjection involves the generation of multicopy extrachromosomal transgene arrays, it is possible that increased dosage of *nol-6* is deleterious during early embryogenesis, resulting in lethality.

### Solexa whole genome sequencing

Sequence sample preparation was performed via the standard Illumina Genome Analyzer genomic sample preparation protocol. In brief, this entails beginning with 5 µg of high quality genomic DNA and fragmenting this DNA via nebulization to sizes of less than 800 bp. The fragment ends are repaired and an ‘A’ base is added to the 3′ ends. Adapters containing a single ‘T’ overhang at their 3′ end are then ligated to the fragments. A fragment size of approximately 200–250 bp is isolated and purified via agarose gel purification. Finally, a short, ten cycle PCR is performed to enrich those DNA fragments that have adapter molecules on both ends and to amplify the amount of DNA in the library without skewing the representation of the library. Following Illumina's standard sequencing protocol, the resultant DNA library was sequenced to a depth of 6× across the entire genome. Solexa genome analyzer single-end reads were produced at a size of 36 base pairs and aligned to the wild-type reference genome (NC_003279-84) at an average depth-coverage of 5×. The data was analyzed using the Mapping and Assembly with Quality (MAQ) software which performs read alignment and SNP prediction [81].

### Construction of *nol-6* RNAi clone

A 197 base pair fragment was amplified using forward primer 5′-tcaggtcgaccattgaaattccgccaaaagc-3′ and reverse primer 5′-tcagggtaccatccaattcgaactccatcg-3′. The fragment was cloned into the SalI and KpnI sites of pL4440 (Open Biosystems) and transformed into *E. coli* HT115(DE3) cells.

### RNAi

We used the RNA interference technique to generate loss-of-function RNAi phenotypes by feeding nematodes with *E. coli* expressing double-stranded RNA that is homologous to a target gene [82],[83]. The *E. coli* strain HT115(DE3) harboring the appropriate vectors was grown in LB broth containing 100 µg/ml ampicillin and 10 µg/ml tetracycline at 37°C overnight. Bacteria were plated onto NGM plates containing 100 µg/ml carbenicillin and 2 mM isopropyl β-D-thiogalactoside (IPTG) and were allowed to grow overnight at 37°C.

For knockdown of *nol-6*, eggs were harvested by treatment of gravid adults with alkaline hypochlorite [84] and synchronized to L1 stage overnight in S-basal buffer. Nematodes were grown on plates containing *E. coli* expressing dsRNA for 4 days at 20°C to gravid adult stage before being transferred to *S. enterica* strain SL1344.

For knockdown of *rps* genes, eggs were harvested by bleaching gravid adult nematodes and synchronized to L1 stage for 22 hours in S-basal buffer. L1 larvae were plated onto NGM plates seeded with *E. coli* strain OP50 and grown for 2 days at 20°C to L4 stage before being transferred to RNAi plates as previously described. Nematodes were fed RNAi expressing bacteria for 24 hours at 20°C before being transferred to *S. enterica* strain SL1344. Bacteria strains expressing double-stranded RNA to inactivate the *C. elegans* genes other than *nol-6* were obtained from Wellcome/Cancer Research (Cambridge, U.K) and Open Biosystems (Huntsville, AL). The identity of all clones was confirmed by sequencing.

### Quantitative Real Time PCR

Gravid adult wild-type nematodes were lysed using a solution of sodium hydroxide and bleach, washed, and the eggs were synchronized for 22 hours in S basal liquid medium at room temperature. Synchronized L1 animals were placed onto NGM plates containing 2 mM IPTG and 100 ug/mL carbenicillin seeded with *E. coli* HT115 expressing double stranded RNA against *nol-6* or empty vector and grown until L4 (5 days at 15°C). The L4 animals were fed *S. enterica* for 24 hours at 25°C and then harvested. The nematodes were collected by washing the plates with M9 buffer, and RNA extracted using Trizol reagent. Genomic DNA was removed by treating the RNA samples with DNase using the DNA-free kit according to manufacturer's instruction (Ambion). qRT-PCR was conducted using the Applied Biosystems TaqmanOne-Step Real-time PCR protocol using SYBR Green fluorescence (Applied Biosystems) on an Applied Biosystems 7900HT real-time PCR machine in 96 well plate format. Fifty nanograms of RNA were used for real-time PCR. Twelve microliter reactions were set-up and performed as outlined by the manufacturer (Applied Biosystems). Gene expression for three independent isolations of *nol-6* RNAi nematodes were compared to vector control nematodes using the comparative Ct method after normalization to *act-1,-3,-4* (pan-actin). Primer sequences are available upon request.

### Statistical analyses

Nematode survival was plotted as a nonlinear regression curve using the PRISM 4.00 computer program. Survival curves are considered significantly different from the control when p<0.05. Prism uses the product limit or Kaplan–Meier method to calculate survival fractions and the logrank test, which is equivalent to the Mantel–Heanszel test, to compare survival curves. In each case, curves represent combined data from at least three independent experiments. Mann-Whitney test and Student's exact t test were used to analyze bacterial accumulation and qRT-PCR results, respectively.

### Microarray analysis

Gravid adult wild-type and *rpa-9* nematodes were lysed using a solution of sodium hydroxide and bleach, washed, and the eggs were synchronized for 22 hours in S basal liquid medium at room temperature. Synchronized L1 animals were placed onto NGM plates seeded with *E. coli* OP50 and grown until L4 (40 hours at 20°C). The L4 animals were exposed to *S. enterica* for 12 hours at 25°C and then harvested by washing the plates with M9 buffer. RNA was extracted using Trizol reagent for two independent isolations for wild type nematodes and three independent experiments for *rpa-9* nematodes. cDNA was generated and hybridized to Affymetrix *C. elegans* Genome Array following the manufacturer's instructions at the Duke Microarray Facility. Detailed protocols are available on the Duke Microarray Facility Web site (http://microarray.genome.duke.edu). GeneSpring Software 9.0 (Agilent Technologies) was used to perform normalizations and fold change analysis. Gene lists for *pmk-1* regulated genes [50], *daf-16* regulated genes [3] and genes misregulated by *cep-1* in response to UV [55] have been described. Briefly, microarrays used for *pmk-1* regulated genes [50] were from Affymetrix while the microarrays used for *daf-16* regulated genes [3] and genes misregulated by *cep-1* in response to UV [55] were custom made. Overall, RNA samples were obtained from synchronized by hypochlorite treatment L1 animals that were grown to young adults or L4-yound adults. Statistical significance of enrichment was determined by using a program for comparing two sets of genes (http://elegans.uky.edu/MA/progs/overlap_stats.html). P values are calculated using a method that is essentially the same as EASE [85]. P values are calculated using an exact hypergeometric probability or its binomial approximation where appropriate, using a jackknife adjustment. Either a Holm-Bonferroni or Bonferroni correction for multiple testing is applied.

### Pharyngeal Invasion Assay

This assay was performed essentially as previously described [2],[59] but with minor modifications. Nematodes were synchronized by treatment of gravid adults with sodium hydroxide and bleach. Synchronized L1 larvae were grown on NGM plates seeded with *E. coli* for 4 days at 20°C. One hundred 1 day old adult hermaphroditic nematodes were placed on lawns of *S. enterica (Smo22)* or *E. coli (DH5α)* expressing GFP for 48 hours at 25°C. Nematodes exhibiting infected pharynxes were quantified using fluorescence microscopy.

## Supporting Information

Figure S1 *S. enterica* bacterial load is reduced in *rpa-9* mutant nematodes. Wild type (red dots) and *rpa-9* (blue dots) nematodes were fed *S. enterica*/GFP for 48 hours and sorted using the COPAS Biosort System. Approximately 400 nematodes were analyzed per condition. p<0.0001. (See Text S1, Supplemental material and methods.)
(0.01 MB PDF)

Figure S2 *rpa-9* mutant nematodes are resistant to killing by *P. aeruginosa* at 20 degrees C and 15 degrees C compared to wild type. (A) *rpa-9* and wild-type animals were fed *P. aeruginosa* (PA14) at 20 degrees C (p<0.0001). (B) *rpa-9* and wild-type animals were fed *P. aeruginosa* (PA14) at 15 degrees C (p<0.0001). For each condition, 75 animals were used.
(0.02 MB PDF)

Figure S3 Microarray results were confirmed via qRT-PCR. (A) Five up-regulated and (B) five down-regulated transcripts were confirmed using qRT-PCR with the same RNA used for the microarray. Data represents the average fold change of two independent RNA isolations. Error bars represent SEM. Due to high sequence similarities between msp and nspa genes, primers could not be designed for individual transcripts. *msp* genes represented are *Y59E9AR.1, Y59E9AR.7, Y59H11AM.1, msp-10, msp-113, msp-19, msp-31, msp-36, msp-38, msp-45, msp-51, msp-53, msp-55, msp-56, msp-57, msp-59, msp-65, msp-76, msp-77, msp-78, msp-79, msp-81*. nspa genes represented are: *nspa-1, nspa-10, nspa-2, nspa-3, nspa-4, nspa-5, nspa-6, nspa-7, nspa-8*, and *nspa-9*.
(0.01 MB PDF)

Figure S4 Mapping and identification of the *rpa-9* mutation. (A) RFLP-SNP analysis was performed using 96 *rpa-9* recombinant progeny which displayed the rpa-9 phenotype. The two most informative recombinants identified the end points of a 109 Kb region (1407386–1516505) on the left arm of chromosome II. (B) Solexa whole genome sequencing identified a single mutation within the mapped region which causes a glycine to glutamic acid substitution in the *C. elegans* gene *nol-6*.
(0.02 MB PDF)

Figure S5 *nol-6* RNAi phenocopies the reduced *S. enterica* bacterial load of *rpa-9* mutants. Wild type (red bars) and *rpa-9* (blue bars) nematodes grown on dsRNA for vector control (solid bars) or *nol-6* RNAi (striped bars) were fed *S. enterica*/GFP for 70 hours and the colony forming units were quantified. Ten nematodes were used for each condition. (See Text S1, Supplemental material and methods.)
(0.01 MB PDF)

Figure S6 *nol-6* RNAi phenocopies the reduced fertility of *rpa-9* mutant nematodes. Wild type and *rpa-9* mutant nematodes grown on dsRNA for vector control *nol-6* RNAi were analyzed for fertility by counting the number of eggs laid for 48 hours. Wild-type vector control vs. Wild-type *nol-6* RNAi p<0.0001, wild type vector control vs. *rpa-9* vector control p<0.0001. n = 17 (wt vector control), n = 50 (wt *nol-6* RNAi and *rpa-9* vector control). (See Text S1, Supplemental material and methods.)
(0.05 MB PDF)

Figure S7 Nucleolar size is enlarged in *rpa-9* mutants during *S. enterica* infection. Wild-type N2 nematodes were exposed to *E. coli* or *S. enterica* for 48 hours. *rpa-9* nematodes were exposed to *S. enterica* for 48 hours. The bar graph shows the measurement of the intestinal nucleoli. N2+*E. coli* vs. N2+*S. enterica*: p = 0.034, N2+*S. enterica* vs. *rpa-9*+*S. enterica*: p = 0.036. N = 8–10. Merged images show nucleoli stained with SYTO 12 (green channel) and the gut autofluorescence (red channel). (See Text S1, Supplemental material and methods.)
(0.15 MB PDF)

Figure S8 *cep-1* is required for full enhanced resistance of *rpa-9* nematodes to *P. aeruginosa*. Wild type and *rpa-9* mutant nematodes grown on dsRNA for vector control or dsRNA for *cep-1* were fed *S. enterica*. Wild type vector vs. *rpa-9* vector: p<0.0001. *rpa-9* vector vs. *rpa-9* cep-1 RNAi: p = 0.0018. Wild type vector vs. *rpa-9;cep-1* RNAi: p<0.0001. For each condition, 60 animals were used.
(0.01 MB PDF)

Figure S9 *pmk-1* is required for full enhanced resistance of *rpa-9* mutant nematodes to *P. aeruginosa*. Wild type nematodes and *rpa-9* nematodes grown on dsRNA for vector control or dsRNA for *pmk-1* were fed *P. aeruginosa*. Wild type vector vs. *pmk-1* RNAi: p<0.0001. *rpa-9* vector vs. *pmk-1* RNAi: p<0.0001. Wild type *pmk-1* RNAi vs. *rpa-9;pmk-1* RNAi: p<0.0001. Wild type vector vs. *rpa-9* vector: p<0.0001. For each condition, 60 animals were used.
(0.02 MB PDF)

Figure S10 *daf-16* is required for full enhanced resistance of *rpa-9* mutant nematodes to *P. aeruginosa*. Wild type and *rpa-9* nematodes grown on dsRNA for vector control or dsRNA for *daf-16* were fed *S. enterica*. Wild type vector vs. *rpa-9* vector: p<0.0001. *rpa-9* vector vs. *daf-16* RNAi: p<0.0001. Wild type vector vs. *daf-16* RNAi: p = 0.4429. *rpa-9* vector vs. *rpa-9 daf-16* RNAi: p<0.0001. For each condition, 60 animals were used.
(0.02 MB PDF)

Figure S11 *sym-1* is required for enhanced resistance of *nol-6* RNAi nematodes to *S. enterica*. Wild type and *sym-1(mn601)* mutant nematodes grown on dsRNA for vector control or dsRNA for *nol-6* were fed *S. enterica*. Wild type vector vs. *nol-6*: p<0.0001. No significant differences were found in any other comparison. For each condition, 60 animals were used.
(0.01 MB PDF)

Table S1 Representation factors for gene sets among RPA-9-regulated genes. The representation factor is the number of overlapping genes divided by the expected number of overlapping genes drawn from the group of RPA-9-regulated genes and the group corresponding to a given gene set. For details, see http://elegans.uky.edu/MA/progs/representation.stats.html. *Genes that are induced at least 2 fold upon UV radiation in a *cep-1*-dependent manner.
(0.02 MB XLS)

Table S2 Expression levels of CEP-1 targets that are upregulated in *rpa-9* mutant*. *Genes that require require CEP-1 for induction upon UV irradiation (Derry et al. 2007. Cell Death Differ 14, 662–670). Shown are the mean±error of expression levels in wild type (n = 2) and *rpa-9* (n = 3) animals. Values from individual probes are shown independently. +Genes that are signature of *C. elegans* response to infection (Wong et al. 2007. Gen Biol 8:R194).
(0.02 MB XLS)

Table S3 Expression levels of CEP-1 targets that are downregulated in rpa-9 mutant*. *Genes that are repressed by *cep-1* for upon UV irradiation were obtained from (Derry et al. 2007. Cell Death Differ 14, 662–670). Shown are the mean±error of expression levels in wild type (n = 2) and *rpa-9* (n = 3) animals. Values from individual probes are shown independently.
(0.03 MB XLS)

Table S4 Inhibition of *nol-6* or *rps* genes by RNAi enhances resistance to *S. enterica* in *glp-4(bn2)* animals. N: Number of independent experiments. n: Total number of animals. The median survival is the time at which half the subjects have died.
(0.02 MB XLS)

Text S1 Supplemental material and methods.
(0.06 MB PDF)
