# Supplementary figures and images for: Nucleolar Proteins Suppress Caenorhabditis elegans Innate Immunity by Inhibiting p53/CEP-1
Source: PLoS Genet. 2009 Sep 18;5(9):e1000657. doi: 10.1371/journal.pgen.1000657 (PMC2734340; doi:10.1371/journal.pgen.1000657)

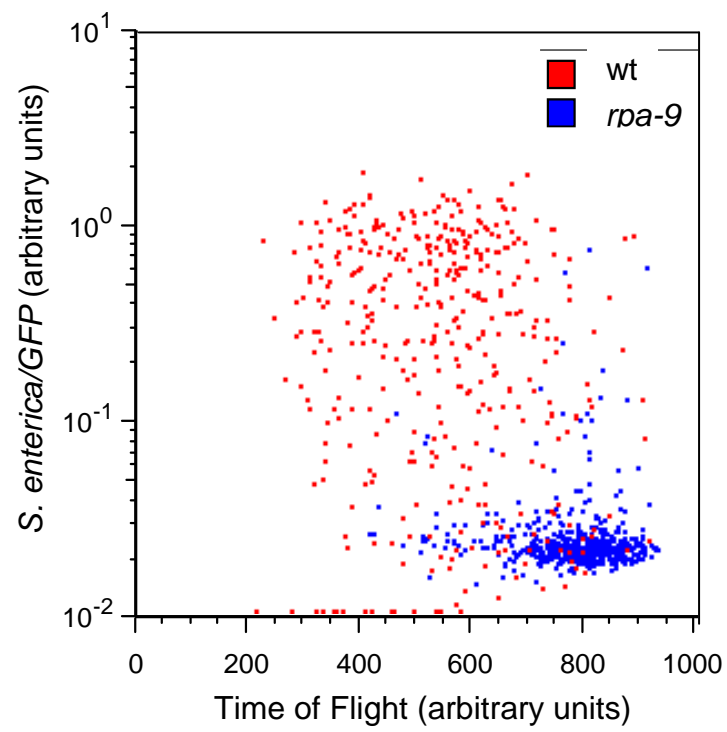

Supplement: Figure S1 — S. enterica bacterial load is reduced in rpa-9 mutant nematodes. Wild type (red dots) and rpa-9 (blue dots) nematodes were fed S. enterica/GFP for 48 hours and sorted using the COPAS Biosort System. Approximately 400 nematodes were analyzed per condition. p<0.0001. (See Text S1, Supplemental material and methods.) (0.01 MB PDF) [file pgen.1000657.s001.pdf]

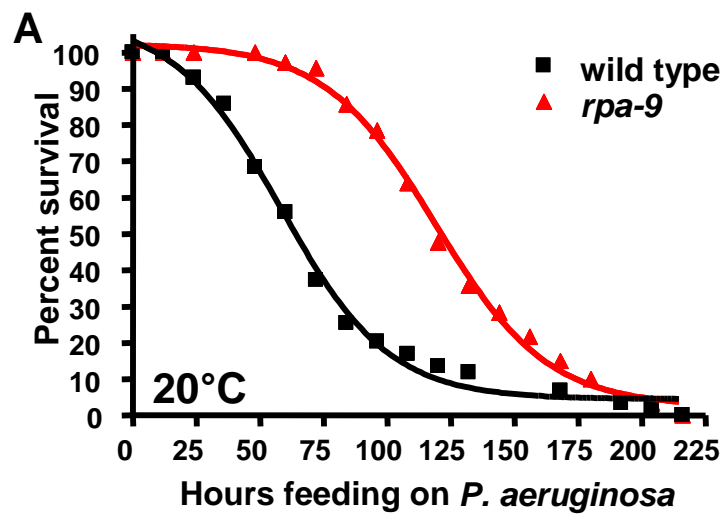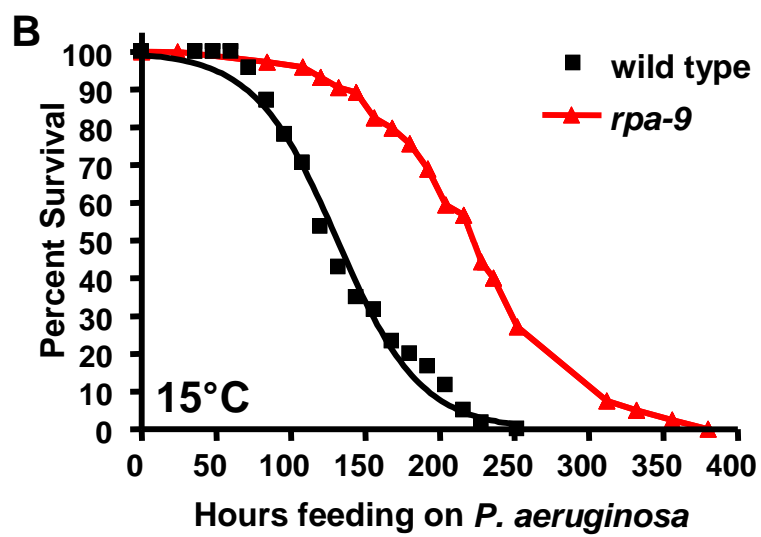

Supplement: Figure S2 — rpa-9 mutant nematodes are resistant to killing by P. aeruginosa at 20 degrees C and 15 degrees C compared to wild type. (A) rpa-9 and wild-type animals were fed P. aeruginosa (PA14) at 20 degrees C (p<0.0001). (B) rpa-9 and wild-type animals were fed P. aeruginosa (PA14) at 15 degrees C (p<0.0001). For each condition, 75 animals were used. (0.02 MB PDF) [file pgen.1000657.s002.pdf]

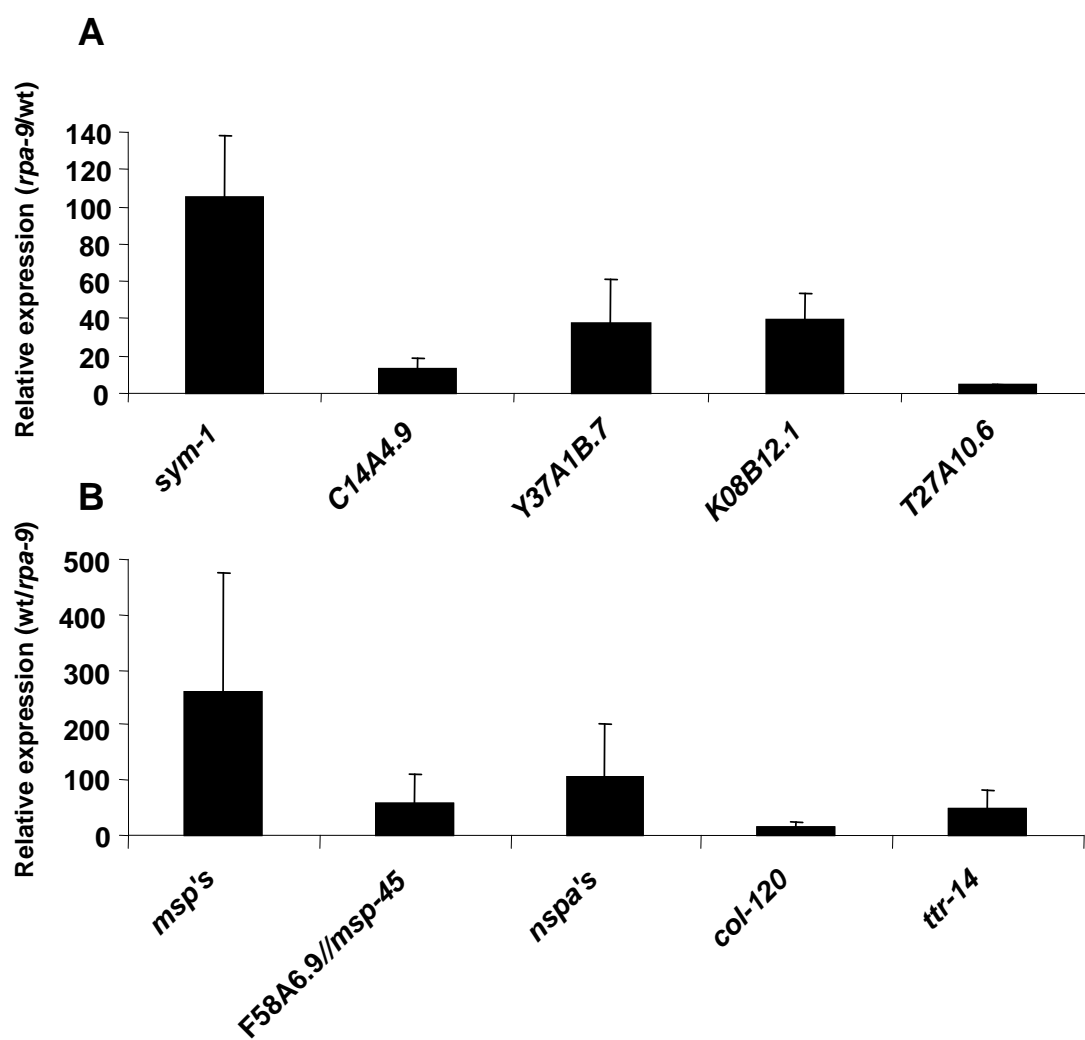

Supplement: Figure S3 — Microarray results were confirmed via qRT-PCR. (A) Five up-regulated and (B) five down-regulated transcripts were confirmed using qRT-PCR with the same RNA used for the microarray. Data represents the average fold change of two independent RNA isolations. Error bars represent SEM. Due to high sequence similarities between msp and nspa genes, primers could not be designed for individual transcripts. msp genes represented are Y59E9AR.1, Y59E9AR.7, Y59H11AM.1, msp-10, msp-113, msp-19, msp-31, msp-36, msp-38, msp-45, msp-51, msp-53, msp-55, msp-56, msp-57, msp-59, msp-65, msp-76, msp-77, msp-78, msp-79, msp-81. nspa genes represented are: nspa-1, nspa-10, nspa-2, nspa-3, nspa-4, nspa-5, nspa-6, nspa-7, nspa-8, and nspa-9. (0.01 MB PDF) [file pgen.1000657.s003.pdf]

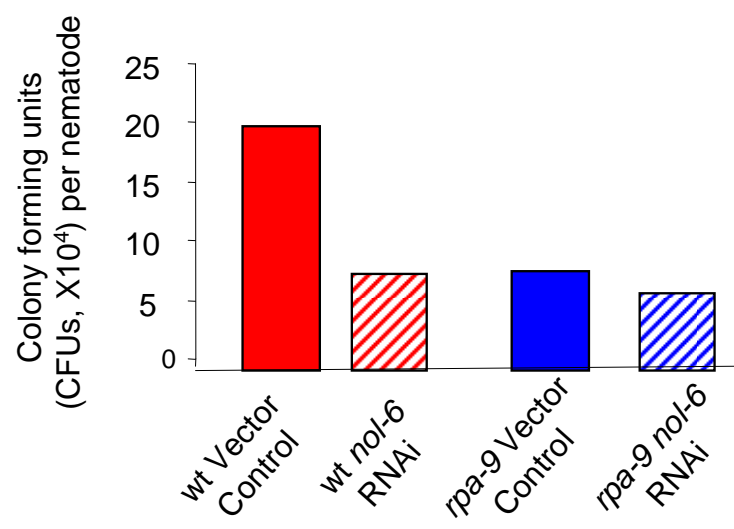

Supplement: Figure S5 — nol-6 RNAi phenocopies the reduced S. enterica bacterial load of rpa-9 mutants. Wild type (red bars) and rpa-9 (blue bars) nematodes grown on dsRNA for vector control (solid bars) or nol-6 RNAi (striped bars) were fed S. enterica/GFP for 70 hours and the colony forming units were quantified. Ten nematodes were used for each condition. (See Text S1, Supplemental material and methods.) (0.01 MB PDF) [file pgen.1000657.s005.pdf]

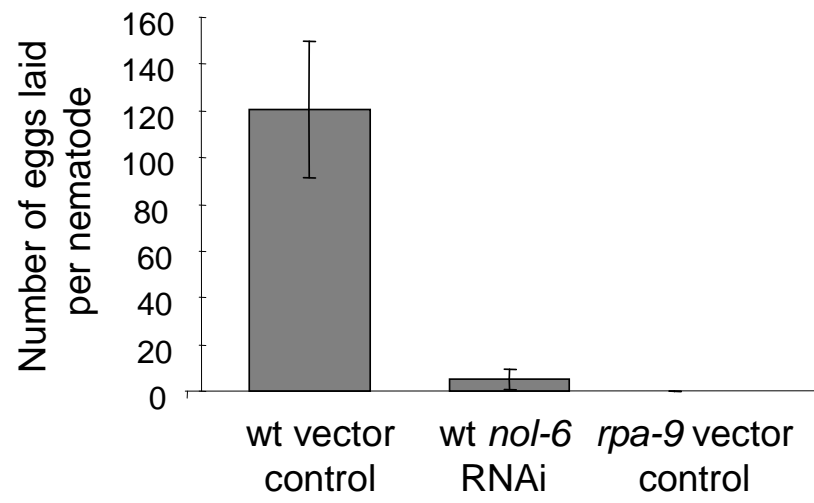

Supplement: Figure S6 — nol-6 RNAi phenocopies the reduced fertility of rpa-9 mutant nematodes. Wild type and rpa-9 mutant nematodes grown on dsRNA for vector control nol-6 RNAi were analyzed for fertility by counting the number of eggs laid for 48 hours. Wild-type vector control vs. Wild-type nol-6 RNAi p<0.0001, wild type vector control vs. rpa-9 vector control p<0.0001. n = 17 (wt vector control), n = 50 (wt nol-6 RNAi and rpa-9 vector control). (See Text S1, Supplemental material and methods.) (0.05 MB PDF) [file pgen.1000657.s006.pdf]

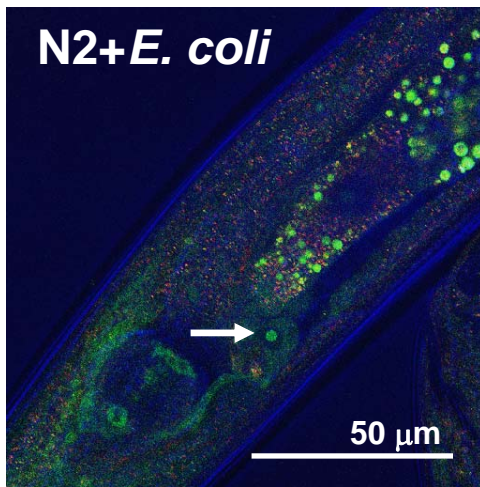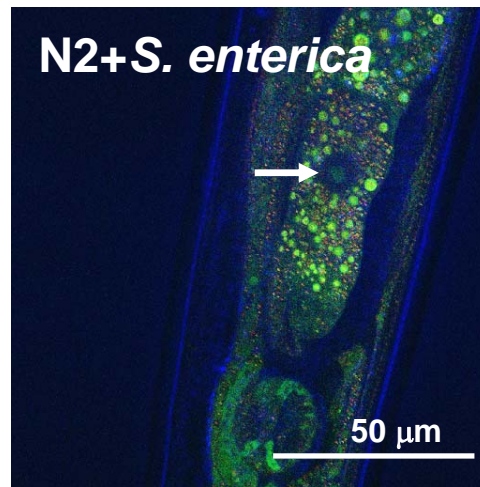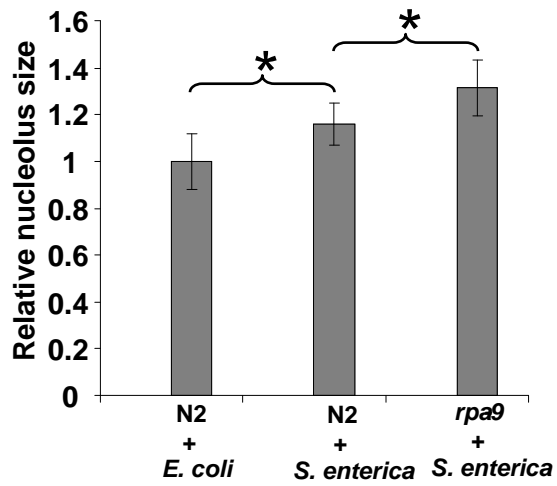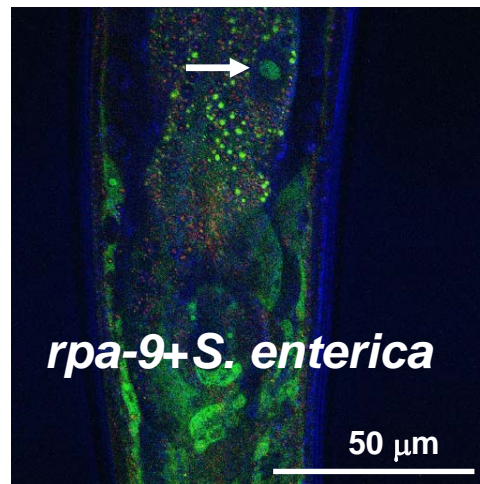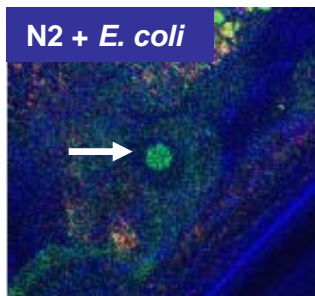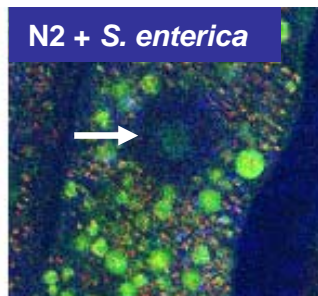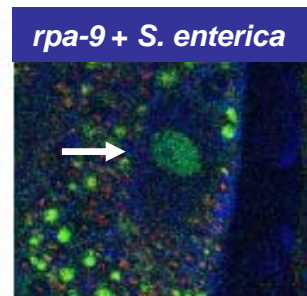

Supplement: Figure S7 — Nucleolar size is enlarged in rpa-9 mutants during S. enterica infection. Wild-type N2 nematodes were exposed to E. coli or S. enterica for 48 hours. rpa-9 nematodes were exposed to S. enterica for 48 hours. The bar graph shows the measurement of the intestinal nucleoli. N2+E. coli vs. N2+S. enterica: p = 0.034, N2+S. enterica vs. rpa-9+S. enterica: p = 0.036. N = 8–10. Merged images show nucleoli stained with SYTO 12 (green channel) and the gut autofluorescence (red channel). (See Text S1, Supplemental material and methods.) (0.15 MB PDF) [file pgen.1000657.s007.pdf]

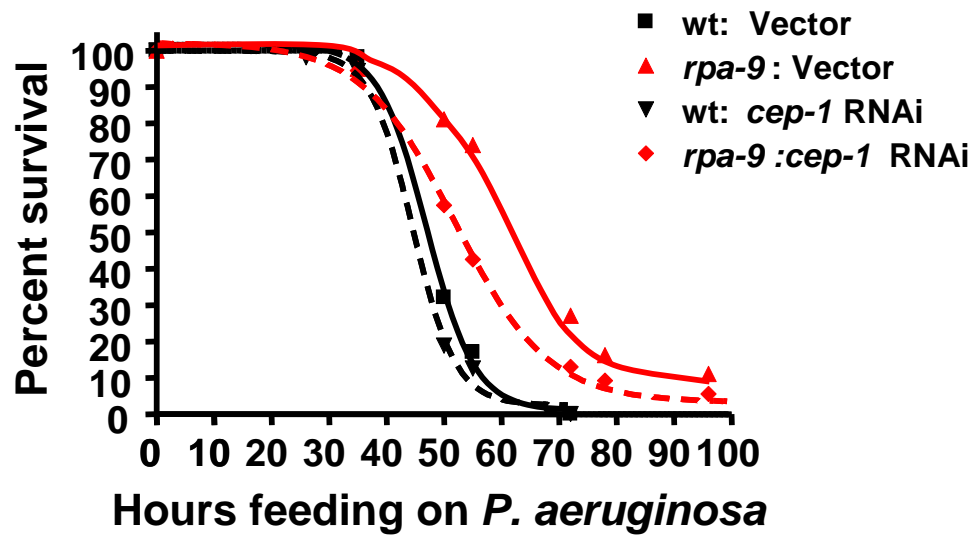

Supplement: Figure S8 — cep-1 is required for full enhanced resistance of rpa-9 nematodes to P. aeruginosa. Wild type and rpa-9 mutant nematodes grown on dsRNA for vector control or dsRNA for cep-1 were fed S. enterica. Wild type vector vs. rpa-9 vector: p<0.0001. rpa-9 vector vs. rpa-9 cep-1 RNAi: p = 0.0018. Wild type vector vs. rpa-9;cep-1 RNAi: p<0.0001. For each condition, 60 animals were used. (0.01 MB PDF) [file pgen.1000657.s008.pdf]

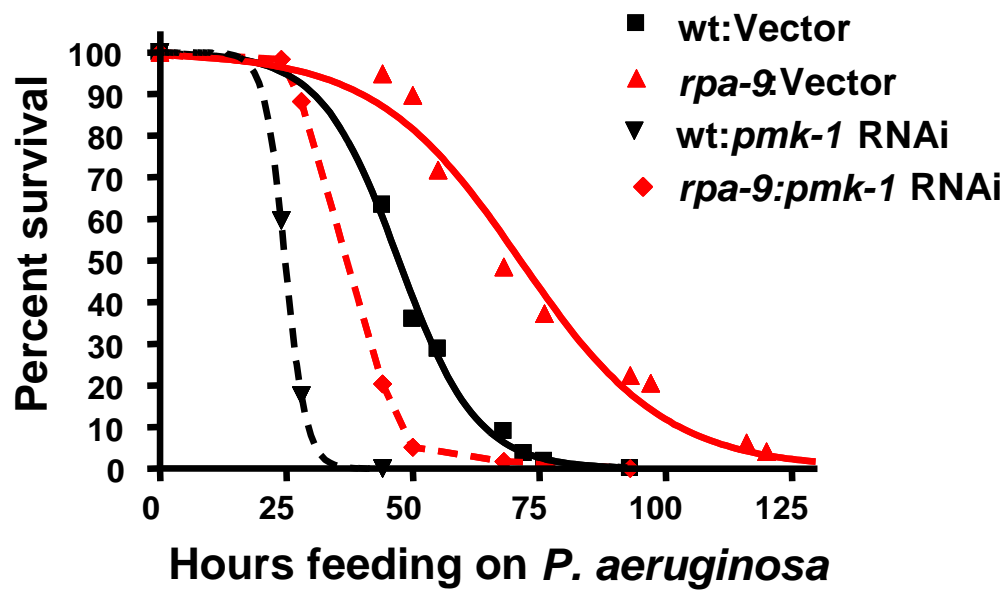

Supplement: Figure S9 — pmk-1 is required for full enhanced resistance of rpa-9 mutant nematodes to P. aeruginosa. Wild type nematodes and rpa-9 nematodes grown on dsRNA for vector control or dsRNA for pmk-1 were fed P. aeruginosa. Wild type vector vs. pmk-1 RNAi: p<0.0001. rpa-9 vector vs. pmk-1 RNAi: p<0.0001. Wild type pmk-1 RNAi vs. rpa-9;pmk-1 RNAi: p<0.0001. Wild type vector vs. rpa-9 vector: p<0.0001. For each condition, 60 animals were used. (0.02 MB PDF) [file pgen.1000657.s009.pdf]

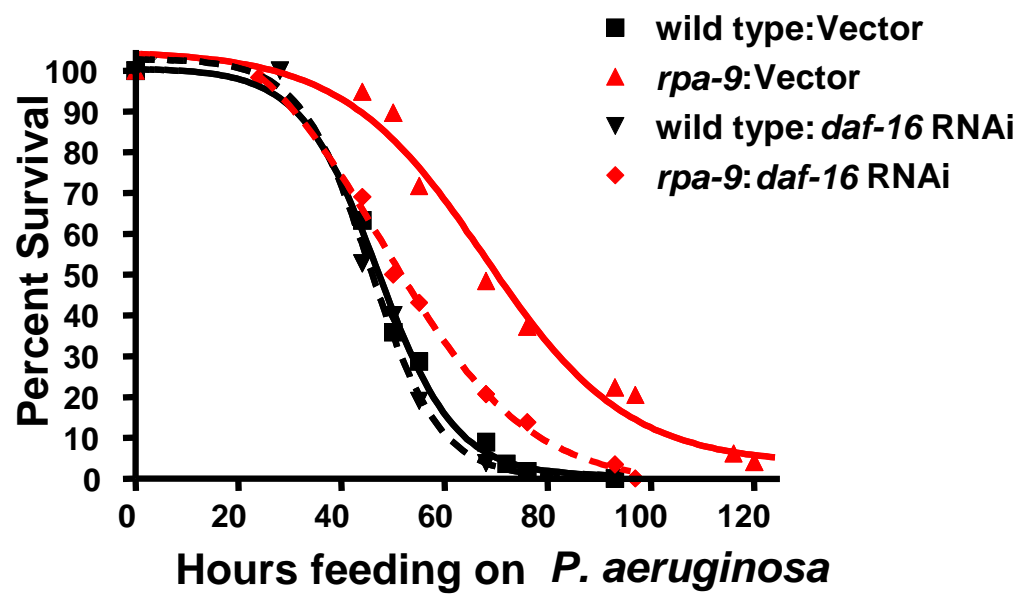

Supplement: Figure S10 — daf-16 is required for full enhanced resistance of rpa-9 mutant nematodes to P. aeruginosa. Wild type and rpa-9 nematodes grown on dsRNA for vector control or dsRNA for daf-16 were fed S. enterica. Wild type vector vs. rpa-9 vector: p<0.0001. rpa-9 vector vs. daf-16 RNAi: p<0.0001. Wild type vector vs. daf-16 RNAi: p = 0.4429. rpa-9 vector vs. rpa-9 daf-16 RNAi: p<0.0001. For each condition, 60 animals were used. (0.02 MB PDF) [file pgen.1000657.s010.pdf]

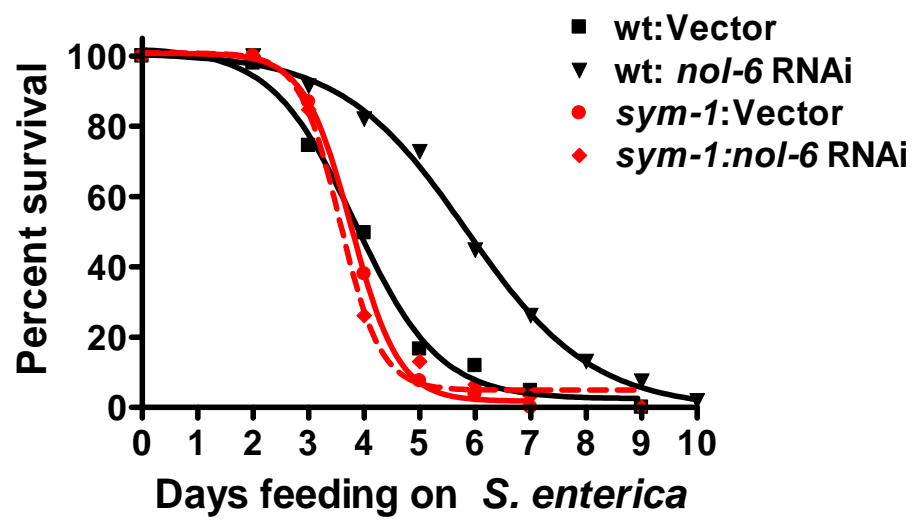

Supplement: Figure S11 — sym-1 is required for enhanced resistance of nol-6 RNAi nematodes to S. enterica. Wild type and sym-1(mn601) mutant nematodes grown on dsRNA for vector control or dsRNA for nol-6 were fed S. enterica. Wild type vector vs. nol-6: p<0.0001. No significant differences were found in any other comparison. For each condition, 60 animals were used. (0.01 MB PDF) [file pgen.1000657.s011.pdf]
