# Supplementary material for: Nucleolar Proteins Suppress Caenorhabditis elegans Innate Immunity by Inhibiting p53/CEP-1
Source: PLoS Genet. 2009 Sep 18;5(9):e1000657. doi: 10.1371/journal.pgen.1000657 (PMC2734340; doi:10.1371/journal.pgen.1000657)

A

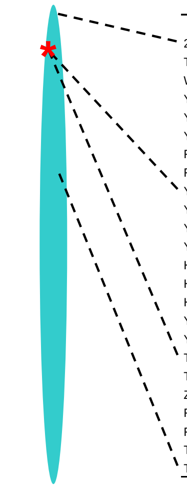

SNP position on Cosmid/YAC clone	Physical Position on Chromosome II	Cross Progeny containing recombination events on the far left end of Chromosome II																									
		1	2	3	4	5	6	7	8	9	10	11	12	13	14	15	16	17	18	19	20	21	22	23	24	25	26
2L52:1962	2,119													H	H												
T01D1:14052	176,721	N	N	N	N	N	N	N	N	N	N	N	N	H	H		N	N	N	N	N	N	N	N	N	N	N
W07E6:20948	492,378													H	H		N										
Y46B2A:12196	862,483													H	H	H	N										
Y57G7A:4213	1,270,098							N	N	N	N	N	N	H	N	H	N		N	N	N	N					
Y57G7A:35700	1,301,585												N	H		N											
F56D12:23102	1,329,402												N	H		N											
F54D12:9666	1,373,022												N	H													
Y51H7C:6136	1,407,386													H													
Y51H7C:11155	1,412,405													N													
Y51H7C:20199	1,421,449													N													
Y51H7C:65463	1,466,763													N													
H17B01:16682	1,486,393												N	N	N	N											
H17B01:22874	1,492,585												N														
H17B01:32151	1,501,862												N														
Y51H7BM:7560	1,516,505												N														
Y51H7BM:7582	1,516,527												N														
T07H3:5408	1,580,643												N														
T07H3:18337	1,593,572												H														
ZC204:4516	1,640,430												H														
F58E1:13123	1,683,954	N	N	N	N	N	N	N	N	N	N	N	H	N	N	N	N	N	N	N	N	N	N	N	N	N	N
F43C11:1220	2,333,130												H	N	N		N										
T10D4:6143	3,122,843	N	N	N	N	N	N	H	H	H	H	H	H	N	N		C	H	H	H	H	H	N	N	N	N	N
T13C2:22236	6,789,143	H	H	H	H	H	H	H	C	C	H	H	H	N	N		C	H	H	H	H	H	C	C	H	H	H

B

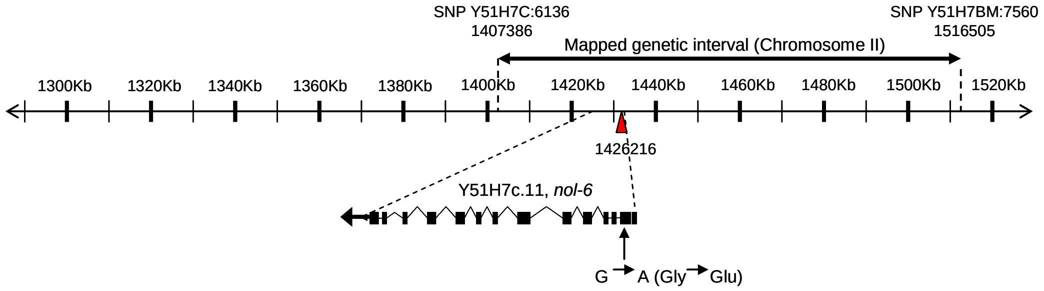

Supplement: Figure S4 — Mapping and identification of the rpa-9 mutation. (A) RFLP-SNP analysis was performed using 96 rpa-9 recombinant progeny which displayed the rpa-9 phenotype. The two most informative recombinants identified the end points of a 109 Kb region (1407386–1516505) on the left arm of chromosome II. (B) Solexa whole genome sequencing identified a single mutation within the mapped region which causes a glycine to glutamic acid substitution in the C. elegans gene nol-6. (0.02 MB PDF) [file pgen.1000657.s004.pdf]
