# Supplementary material for: Nucleolar Proteins Suppress Caenorhabditis elegans Innate Immunity by Inhibiting p53/CEP-1
Source: PLoS Genet. 2009 Sep 18;5(9):e1000657. doi: 10.1371/journal.pgen.1000657 (PMC2734340; doi:10.1371/journal.pgen.1000657)
Supplement: Text S1 — Supplemental material and methods. (0.06 MB PDF) [file pgen.1000657.s016.pdf]

Supplemental Material and Methods

Confocal microscopy

Wild type and *rpa-9* mutants were synchronized by treatment of gravid adults with sodium hydroxide and bleach. L1 larvae were grown on *E. coli* carrying an empty vector for 4 days at 20˚C before being transferred to plates seeded with *S. enterica* or *E. coli* for 48 hours at 25˚C. To quantify the nucleoli size, the animals were stained with SYTO 12. Briefly, the animals were incubated in 50 µM SYTO 12 for 3 h at room temperature. Twenty nematodes were anesthetized in 10% sodium azide on an agar pad (2% agarose) and examined using a Leica TCS SL confocal microscope with Leica Confocal software version 2.61 Build 1537 (Leica Microsystems Heidelberg GmbH). The images were printed and the nucleoli were measured at the widest diameter for all conditions and normalized to the control (wild type animals grown on *E. coli*).

Quantification of intestinal *S. enterica* loads

For the quantification of colony forming units, wild type and *rpa-9* nematodes were synchronized by treatment of gravid adults with sodium hydroxide and bleach. Synchronized L1 larvae were grown on dsRNA expressing *nol-6* or empty vector for 4 days at 20˚C before being transferred to plates seeded with *S. enterica/GFP* for 70 hours at 25˚C. Nematodes were transferred to an NGM plate seeded *with E. coli* for 15 to eliminate *S. enterica* stuck to the body of the worms. Nematodes were transferred to a new NGM plate seeded with *E. coli* for one hour to further eliminate external *S. enterica* cells. Ten nematodes per condition were transferred into 50 microliters PBS plus 0.1% Triton and ground. Serial dilutions of the lysates (10^{-1}, 10^{-2}, 10^{-3}, 10^{-4}) were plated onto LB/amp to select for *S. enterica/GFP* cells and grown for 24 hours at 37˚C.

For quantification of *S. enterica/GFP* using the COPAS Biosort system, nematodes were synchronized by treatment of gravid adults with sodium hydroxide and bleach. Synchronized L1 larvae were grown on NGM plates seeded with *E. coli* for 4 days at 20˚C before being transferred to plates seeded with *S. enterica/GFP* for 48 hours at 25˚C. Nematodes were harvested in M9 buffer, washed in M9 three times for ten minutes and resuspended in 30 mL S-basal buffer for 30 minutes to remove any external bacterial cells not persistently attached to the *C. elegans* intestine. Nematodes were sorted using the COPAS Biosort (Union Biometrica) system which reports time of flight (TOF; nematode length, measured in arbitrary COPAS Biosort units) and green fluorescence (GFP-induced fluorescence, measured in arbitrary units) for each individual animal. Results were graphed and analyzed using FlowJo 7.5 software. Significance values were determined using Student's T-Test in Graphpad Prism 4.0.

Fertility Assay

Wild type, *nol-6* RNAi and *rpa-9* nematodes were synchronized by treatment of gravid adults with sodium hydroxide and bleach. Synchronized L1 larvae were grown on NGM plates seeded with *E. coli* for 2 days at 25˚C. Young adult nematodes were individually transferred to separate NGM plates seeded with *E. coli* for 2 days at 25˚C with daily transfers. The plates were incubated at 25˚C for 24 hours and the number of larvae was quantified for each condition.
